# The Effects of Smoking on Respiratory Microbiota Diversity and Composition: A Systematic Review with Exploratory Meta-Analysis

**DOI:** 10.3390/microorganisms14081688

**Published:** 2026-08-01

**Authors:** Ali El Tawil, Joelle Korban, Hassan Abbas, Imad Al Kaakour, Fayez Yassine, Melhem Bilen

**Affiliations:** 1 Department of Experimental Pathology, Immunology and Microbiology, Faculty of Medicine, American University of Beirut, Beirut 1107 2020, Lebanon; ahe37@mail.aub.edu (A.E.T.); jek22@mail.aub.edu (J.K.); hka28@mail.aub.edu (H.A.); ina02@mail.aub.edu (I.A.K.); fky02@mail.aub.edu (F.Y.); 2 Center for Infectious Diseases Research, American University of Beirut, Beirut 1107 2020, Lebanon; 3 World Health Organization (WHO) Collaborating Center for Reference and Research on Bacterial Pathogens, Beirut 1107 2020, Lebanon

**Keywords:** respiratory microbiota, smoking, dysbiosis, upper airways, lower airways, 16S rRNA sequencing, systematic review, meta-analysis

## Abstract

Smoking is a major modifier of respiratory health, but its effects on the respiratory microbiota remain incompletely defined across airway niches and exposure types. We conducted a PRISMA-guided systematic review of PubMed, Web of Science, and Scopus, to identify human studies using culture-independent sequencing to examine associations between smoking and microbiota composition and diversity in anatomically defined upper and lower airway sites. Thirteen studies met the inclusion criteria. The exploratory meta-analysis of bronchoalveolar lavage inverse Simpson diversity from two studies did not provide a precise pooled estimate between smoking and diversity and was highly heterogeneous (SMD −1.63, 95% CI −5.07 to 1.81; I^2^ = 96%). Across studies, smoking was more often associated with reduced alpha diversity. In the oropharynx, smokers more consistently showed higher abundance of *Streptococcus*, *Veillonella*, *Actinomyces*, and *Atopobium*, with lower abundance of *Neisseria* and other oral commensals. In the lower airways, smoking was more often associated with reduced *Prevotella* and *Veillonella*. Nasal and nasopharyngeal findings were smaller and more variable. Overall, current evidence suggests site-specific smoking-associated respiratory microbiota remodeling. However, precise pooled estimates remain limited by substantial inter-study heterogeneity. Therefore, the meta-analysis should be interpreted as exploratory and hypothesis-generating.

## 1. Introduction

The human body harbors diverse microbial communities that contribute to health, and shifts in microbial composition have been associated with a wide range of diseases [1]. Although the lung was historically considered sterile, culture-independent DNA sequencing has demonstrated that healthy lungs contain resident microbial communities [2].

The respiratory microbiota contributes to pulmonary homeostasis by supporting immune tolerance and shaping antigen-presenting cell and regulatory T-cell responses, while also enhancing host defenses through immune priming and competitive exclusion [3]. They further support mucosal barrier integrity via interactions with airway epithelium that influence barrier structure, mucus production, and mucociliary clearance [4].

Airway community composition reflects a dynamic balance between microbial immigration, elimination, and selective pressures imposed by host and environmental factors [5]. Respiratory microbial communities are spatially organized along the airway tract, with distinct but interconnected profiles across nasal, nasopharyngeal, oropharyngeal, laryngeal, and lower-airway niches [6,7]. Upper-airway sites are generally more heavily colonized and shaped by direct environmental exposure, whereas lower-airway communities are lower in biomass and influenced by microbial immigration from upper-airway reservoirs, host clearance mechanisms, and local airway conditions [5,6,8].

Respiratory microbiota have been characterized using culture-independent methods, including 16S rRNA gene sequencing, metagenomic sequencing, and quantitative microbial profiling, across specimens such as oral/oropharyngeal swabs, nasal and nasopharyngeal samples, sputum, bronchoalveolar lavage, bronchial brushings, and lung tissue [9,10]. These approaches have shown that respiratory dysbiosis differs by disease and anatomical site [9]. In chronic obstructive pulmonary disease, airway communities often show reduced diversity and enrichment of Proteobacteria, particularly *Haemophilus*, *Moraxella*, and *Pseudomonas*, with these shifts becoming more pronounced during exacerbations and in neutrophilic inflammatory phenotypes [11,12,13]. In asthma, lower-airway and upper-airway studies have reported enrichment of Proteobacteria, including *Haemophilus* and *Moraxella*, as well as altered *Streptococcus*, *Prevotella*, and *Veillonella* abundance, with associations with airway inflammation, corticosteroid response, and disease severity [14,15]. During respiratory infections, microbial communities may lose stability and become dominated by pathogenic taxa, reflecting reduced colonization resistance and impaired community resilience [9,16,17].

The impact of smoking on the respiratory microbiome may differ according to smoking modality. Combustible cigarette smoke exposes the respiratory tract to nicotine, tar, oxidants, and combustion-derived toxicants that can impair epithelial barrier integrity, mucociliary clearance, and innate immune responses, thereby creating selective pressures that favor dysbiosis [5,18]. Studies comparing smokers and non-smokers have reported smoking-associated shifts in microbial diversity and relative abundance patterns, including enrichment of potentially pathogenic or inflammatory taxa and depletion of health-associated commensals, although findings vary across oral, upper-airway, sputum, and lower-airway samples [19,20]. These changes may be further amplified by smoking-induced immune dysregulation, including impaired macrophage and neutrophil function and heightened inflammatory signaling, which weakens host control of microbial communities [18]. In contrast, electronic cigarettes and vaping devices deliver aerosolized nicotine, solvents, flavoring agents, aldehydes, and metals without tobacco combustion, suggesting that their microbiome effects may overlap with, but not fully mirror, those of conventional cigarettes [21]. Available studies suggest that e-cigarette use can alter oral and airway microbial composition and may increase susceptibility to microbial colonization or infection [22,23]. Evidence for heated tobacco products and other smoking modalities remains more limited, highlighting the need to interpret smoking-associated microbiota changes according to exposure type, duration, intensity, and sampled airway site [24].

Because the included studies sampled oral, oropharyngeal, nasopharyngeal, nasal, laryngeal, and lower-airway sites, this review uses the broader term “respiratory microbiota.” This terminology is supported by the oral–lung axis, which refers to the ecological and immunological connection between microbial communities of the oral cavity/oropharynx and those of the lower respiratory tract [25]. In this axis, the oral cavity and upper airways may serve as reservoirs for microbes that reach the lower airways through micro-aspiration [7,8]. Because the lower respiratory tract is a low-biomass microbial environment, even low-level microbial immigration from oral and upper-airway sites may influence lower-airway community composition [6,8]. Lower-airway communities are further shaped by the balance between microbial immigration, microbial elimination through cough and mucociliary clearance, and local airway growth conditions [5]. Therefore, oral and upper-airway samples were considered relevant to respiratory microbial ecology, while results were interpreted according to the specific anatomical site sampled [5,6,7,8].

Taken together, these anatomical and ecological connections suggest that smoking-associated airway microbiota changes may be relevant to respiratory disease susceptibility [26]. Dysbiosis characterized by reduced microbial diversity and enrichment of oral-derived anaerobes has been observed in chronic obstructive pulmonary disease, where airway microbial dysbiosis has been associated with inflammation and disease progression [27]. Similar microbial signatures have also been highlighted in lung cancer, with smoking-related taxa shifts toward pro-tumorigenic environments [28]. Despite growing evidence of microbiota differences between smokers and non-smokers, findings remain heterogeneous across cohorts and airway sites. We therefore conducted a systematic review to synthesize available human data on respiratory microbiota diversity and composition in relation to smoking and to assess whether consistent, site-specific smoking signatures emerge across studies.

## 2. Methods

This systematic review was conducted following the Preferred Reporting Items for Systematic reviews and Meta-Analyses (PRISMA) guidelines [29]. A PRISMA 2020 checklist was filled out (Supplementary Material S1). The systematic review protocol was not prospectively registered.

### 2.1. Search Strategy

A comprehensive literature search was performed using PubMed, Web of Science and Scopus databases from database inception to 25 July 2025. MeSH terms related to smoking, respiratory and microbiota were used to retrieve relevant studies. The queries used for each database can be found in Supplementary Material S2.

### 2.2. Study Eligibility Criteria

The inclusion criteria required articles to be original research, and focused on “smoking,” “respiratory microbiota” or their respective MeSH terms. We used the PICO model to determine the eligibility criteria. Only studies involving human participants were deemed eligible for inclusion. No language exclusions were applied. Studies were excluded if they were reviews or other forms of non-original research (editorials, case reports, etc.) or animal models. No time limit was set for extracting studies. Article-type and human-study eligibility criteria were applied during title/abstract and full-text screening instead of database-specific search filters. Moreover, anatomically defined respiratory and airway-adjacent locations relevant to the oral–lung axis were used to determine what fell under the respiratory system and what did not. In our literature search we included any of the articles that studied the following: nasal cavity, oral/oropharynx, nasopharynx, larynx, and lower airways/lung samples. Under the term “Smoking” we have included all the studies that discussed cigarette smoking, hookah smoking, waterpipe smoking, Electronic Nicotine Delivery Systems (ENDS) such as E-cigarettes, vapes, electrically heated tobacco products, etc. In this review, “ENDS/e-cigarette use” refers to Electronic Nicotine Delivery Systems, including e-cigarettes and vaping devices, unless an original study used a more specific exposure definition. Passive smoke exposure/environmental tobacco smoke (ETS) was not defined as a separate a priori exposure category. The primary focus was on active smoking and smoking-related products. Studies that evaluated tobacco-smoke exposure using passive exposure definitions or biochemical exposure markers were considered eligible when they otherwise met the review criteria including human participants, culture-independent microbiota assessment, and anatomically defined respiratory sampling sites. The reference lists of the included studies were not systematically searched. Gray literature and preprints were not systematically searched, and only full-text published studies were included.

### 2.3. Study Screening and Selection

The screening process involved two independent reviewers (A.E.T. and F.Y.) who assessed the eligibility of the studies. Any disagreements were resolved by a third reviewer (H.A.). The initial screening was based on the titles and abstracts of the articles, followed by a full-text review of the selected studies. The “Rayyan.AI” tool (Rayyan Systems Inc., Cambridge, MA, USA) was used to organize references and remove duplicates [30] (https://www.rayyan.ai/). Duplicate records were identified and removed using Rayyan’s duplicate-detection function. It was not used to make final inclusion or exclusion decisions.

### 2.4. Data Extraction and Variables

Data extraction was conducted and revised by three authors (A.E.T., J.K. and H.A.), who subsequently validated the data to ensure accuracy. The data extracted from each study included: the primary author, year of publication, the country where the study was conducted, sample size, sex composition, and the technique used to analyze the respiratory microbiota. These data were recorded in a table (Table S1, Supplementary Material S3).

Additionally, data on alpha-diversity and richness metrics (Shannon, Simpson, Chao1, etc.) were extracted, along with beta diversity, exclusion criteria of each study, specimen type, the respiratory site, abundance of taxa at the phylum, genus, and species levels. Any significant alterations in microbial composition among smokers or smoking-related exposure groups relative to non-smoking, never-smoking, or otherwise defined comparison groups were also documented. The extracted data were summarized in a table (Table S2, Supplementary Material S3). The taxa abundances were reported in a spreadsheet (Supplementary Material S4).

### 2.5. Methodological Quality Assessment

All studies were graded via the Methodological index for Non-randomized Studies (MINORS), which has been previously used in systematic reviews and meta-analyses [31]. This was done by 3 reviewers. MINORS was used to evaluate the general methodological quality of non-randomized studies. The MINORS scale differentiates between comparative and non-comparative studies, scoring comparative studies over 24 points and non-comparative studies, scoring out of 16 points. Each item on the scale was scored from 0 to 2 points, assessing various quality measures. Studies were then grouped into quartiles based on their scores. For comparative studies, the first quartile ranged from 0 to 6, the second from 7 to 12, the third from 13 to 18 and the fourth from 19 to 24. Non-comparative studies were grouped into quartiles ranging from 1 to 4 points for the first quartile, 5 to 8 for the second, 9 to 12 for the third, and 13 to 16 for the fourth quartile.

### 2.6. Risk of Bias Assessment

Risk of bias assessment was performed using the Joanna Briggs Institute (JBI) critical appraisal checklist for analytical cross-sectional studies [32]. This tool was chosen because the included studies primarily used cross-sectional observational designs to study associations between smoking or e-cigarette exposure and respiratory microbiota. Each study was evaluated across eight domains like eligibility criteria, description of participants and setting, exposure measurement, outcome measurement, identification and handling of confounders, and statistical analysis. Responses were recorded as “Yes,” “No,” “Unclear,” or “Not applicable.” An overall appraisal decision was assigned for each study. This assessment was done by two reviewers, and any disagreements were resolved by a third reviewer. The JBI checklist can be seen in Supplementary Material S5.

MINORS and JBI checklist were used as complementary appraisal tools, not as interchangeable tools. MINORS provided a standardized assessment of the overall methodological quality of non-randomized studies whereas the JBI checklist allowed additional appraisal of study design-specific risk of bias domains (participant selection, exposure and outcome measurement, identification and handling of confounders, and appropriateness of statistical analysis). The assessments’ results were used to contextualize the strength and reliability of the evidence. They were also used to guide cautious interpretation of findings rather than to exclude studies solely based on quality score.

### 2.7. Microbiome-Specific Methodological Assessment

We performed an additional narrative microbiome-specific methodological assessment because MINORS and JBI do not fully capture technical sources of bias specific to microbiome studies. We summarized specimen type, sequencing region and platform, contamination-control reporting, low-biomass considerations, bioinformatic pipeline, contaminant filtering, and read-depth or rarefaction thresholds where available. This assessment was used to contextualize interpretation of taxonomic and diversity findings rather than to exclude studies or generate a formal quality score.

### 2.8. Quantitative Analysis

A meta-analysis was conducted where sufficiently comparable data were available. Due to heterogeneity in the alpha diversity metrics utilized, airway niche studied, and specimens collected, only two studies qualified for meta-analysis. The meta-analysis was performed using standardized mean differences (Hedges g) with a random-effects model. Statistical heterogeneity was assessed using Cochrane’s Q and the I^2^ statistic. Forest plots were generated using Review Manager Web (RevMan Web, version 11.0.0; The Cochrane Collaboration, London, UK) [33]. Since only two BAL studies were comparable for quantitative synthesis, this meta-analysis was planned and interpreted as exploratory and hypothesis-generating.

### 2.9. Qualitative Analysis

The outcomes that were not suitable for meta-analysis (beta diversity and taxonomic composition) were synthesized narratively and presented as the reported direction of effect. Therefore, findings were reported as higher or lower diversity, community separation, or higher or lower relative abundance in smokers compared with non-smokers. Studies were considered eligible for each synthesis based on comparability of outcome type, airway niche, specimen type, and reported diversity of taxonomic metrics. Moreover, potential sources of heterogeneity were explored descriptively by comparing findings across airway niche, specimen type, and alpha-diversity metrics.

## 3. Results

### 3.1. Included Study Characteristics and Quality Summary

**Figure 1:**
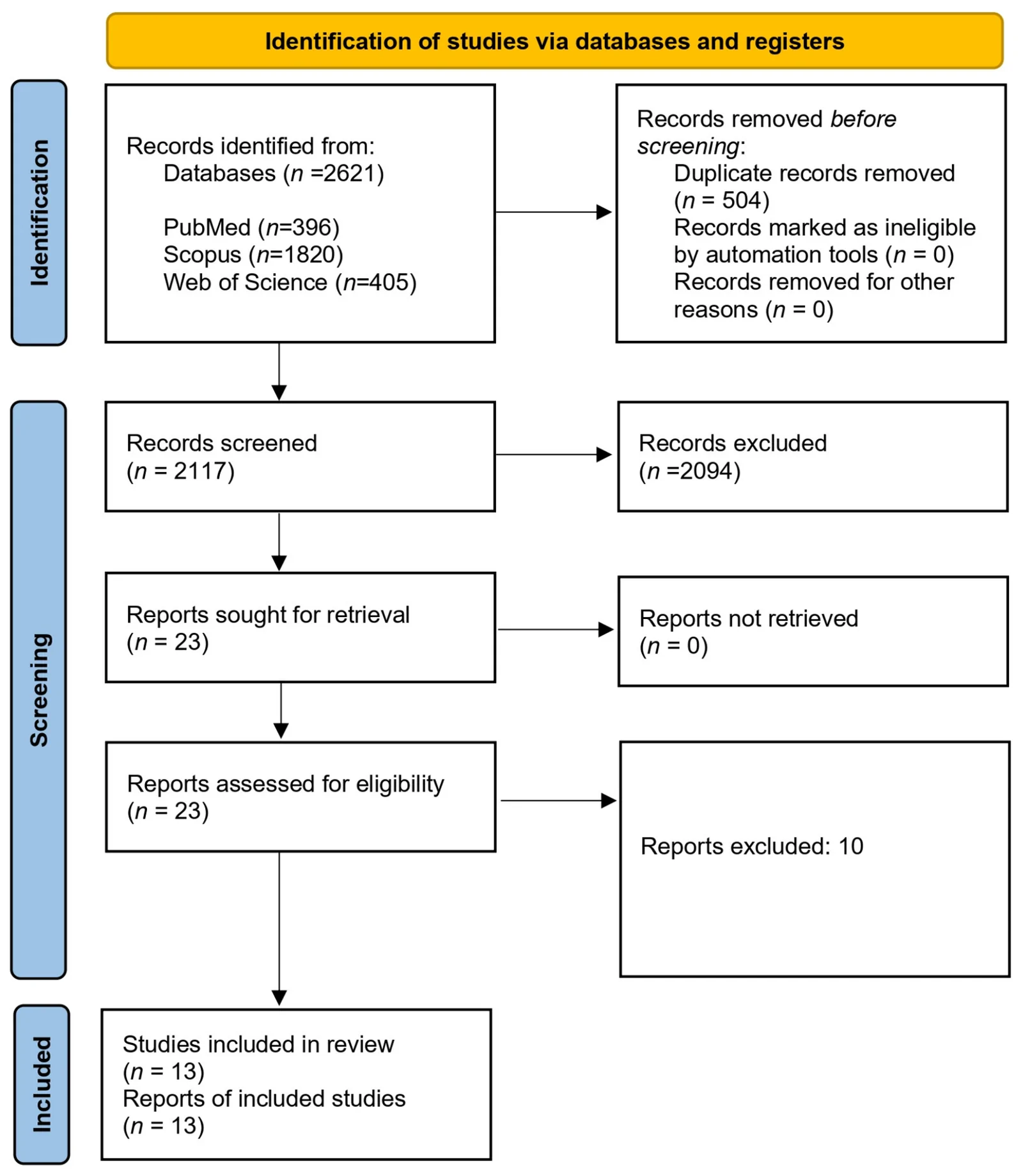
PRISMA 2020 flow diagram of study selection. Databases were searched to find the records (PubMed *n* = 396; Scopus *n* = 1820; Web of Science *n* = 405; total *n* = 2621). A total of 2117 papers were screened after duplicates (*n* = 504) were discarded. After title/abstract screening, 23 full-text articles were evaluated for eligibility. 13 studies were added to the systematic review, while 10 were filtered out.

The initial search across PubMed, Scopus, and Web of Science databases identified 2621 articles. After removing 504 duplicates, 2094 articles were excluded for not being relevant to the target topic. A further 10 were excluded for not meeting the eligibility criteria during the full-text review, resulting in 13 studies that were ultimately included in the systematic review. The excluded studies and reasons for exclusion are provided in Supplementary Material S6. The PRISMA flow diagram (Figure 1) outlines the study selection process.

#### 3.1.1. Study Quality and Risk of Bias Assessment (MINORS and JBI)

All 13 articles were graded via the MINORS scale, appropriate for the observational nature of the included studies (Table S3, Supplementary Material S3). The overall mean MINORS score was 14.9 ± 1.2, ranging from 14 to 18.

Based on the JBI checklist, the overall risk of bias across studies was considered low to moderate. Most studies clearly met most of the domains. The most common limitation was unclear exposure measurement as smoking or e-cigarette status was often based on self-report without biochemical confirmation. In addition, some studies also had unclear or limited control for potential confounders like demographic differences, antibiotic exposure, and clinical characteristics.

#### 3.1.2. Microbiome-Specific Methodological Reporting

Among the core articles, microbiome-specific methodological reporting showed variations. Most studies reported the sampled airway site, sequencing target, and sequencing platform. The targeted 16S region and sequencing approach differed, including V1–V2, V1–V3/V3–V5, V2–V9, V3–V4, V4, V6–V8, and metatranscriptomic or metagenomic approaches. Also, there was heterogeneity in the bioinformatic pipelines. The pipelines across the studies included QIIME-based workflows, mothur, DADA2, UPARSE, Kraken/Bracken, decontam-based contaminant filtering, and other study-specific approaches.

Contamination controls were inconsistently reported. Several studies noted negative, reagent, extraction, PCR, mock-community, or procedural controls whereas others did not clearly report negative or reagent controls. Low-biomass concerns were most relevant for BAL, BALF, and laryngeal tissue studies. Some lower airway studies addressed these concerns through procedural controls, control-sample comparison, contaminant filtering, or discussion of bronchoscope/upper-airway carryover. By contrast, other BAL/BALF studies did not clearly report negative controls or contaminant-filtering procedures. As such, lower-airway taxonomic findings, particularly those involving environmental or water-associated organisms, were interpreted cautiously. A study-level summary of microbiome-specific methodological features is provided in Supplementary Material S7.

#### 3.1.3. Control Group Definition and Baseline Characteristics

The characteristics of the control groups across the 13 core studies were summarized in Supplementary Material S3, Table S1. The comparison groups were composed of adult participants, most often defined as non-smokers or never-smokers, although the health status and recruitment setting varied across studies. When it came to age, studies recruited healthy adults aged 18–40 [34] or 18–50 years [35], while others focused on slightly older populations (≥40 years) [36]. Other studies included broader adult age bands (e.g., 21–65 years [37], 18–80 years [20], or a mean age of ~56 years [38]. These data suggest that the comparison groups generally represented young to older adults rather than pediatric or very elderly populations. Sex distributions were usually mixed and sometimes explicitly described as balanced by age, sex, and race [39]. One study reported a modest female predominance (~56% female among primarily nonsmoking volunteers) [37].

Comparison participants were described as “healthy” or “asymptomatic” in many studies, but this terminology was not uniform across all cohorts and should be interpreted in relation to each study’s recruitment setting and exclusion criteria. Studies excluded individuals with chronic respiratory or systemic diseases unless they were subject to study in their experiment like in [38]. Multiple studies excluded participants based on acute or chronic respiratory illnesses [20,37,40], but the period varied from one study to the next. One study specifically excluded cardiorespiratory disease [35]. Other exclusions included recent influenza vaccination [40], or recent antibiotic use, with the ranges varying per study. Two studies added restrictions on individuals using immunomodulators [20,37]. Some studies restricted participant inclusion based on surgeries performed [35,37]. One study solely studied patients undergoing bronchoscopy [41]. Two studies excluded participants with cancer [42,43]. One study noted the exclusion of individuals exposed to head/neck radiation [37] while another explicitly excluded pregnant women [35].

Smoking status definitions in the control groups were generally stringent. Controls were labeled differently as “nonsmokers,” “non-smokers” or “never-smokers.” When criteria were specified, they included very low lifetime cigarette exposure and no recent smoking (e.g., <100 lifetime cigarettes [39,42,43]), or no smoking within the prior year for those classified as nonsmokers [37]. In multigroup designs that included current smokers, ex-smokers, e-cigarette users, or disease exposure, the control group was always the non-smoking or never-smoking category. Some studies additionally characterized smoking exposure among non-control groups in pack-years or by intensity categories (e.g., mild vs. heavy smokers [44]), reinforcing that the control participants represented the lowest-exposure reference group.

Overall, these characteristics suggest that the comparison groups across studies can be characterized as predominantly adult non-smokers or never-smokers, typically free of major respiratory comorbidities and recent antibiotics usage, although not all comparison groups should be interpreted as uniformly healthy. For this reason, we use the terms “comparison participants,” “non-smokers,” or “never-smokers” throughout the manuscript and reserve “healthy” only for studies that explicitly described their comparison group as such.

#### 3.1.4. Smoker/Exposure Group Definitions and Baseline Characteristics

The smoking-related exposure groups were mostly made up of adult participants with current or past exposures to combustible cigarettes or other-smoking-related exposures, depending on the study (Supplementary Material S3, Table S1). The majority of studies included current cigarette smokers, in addition to at least one comparison group like never-smokers, ex-smokers, or users of electronic cigarettes (ECs). The size of the groups varied. Some cohorts included small samples like three smokers among 10 healthy volunteers in [40]. Other cohorts were larger with 29 smokers in [43], 19 smokers in [20] and 60 current smokers in [38]. EC user groups were included in [35] (24 EC users in the microbiome analysis) and [39] (10 EC users). Other exposure-defined groups included pneumococcal carriers versus non-carriers (12 vs. 47 adults, respectively) with concurrent smoking stratification [45]. One cohort studied adults classified by active versus passive smoke exposure using urinary nicotine metabolites [44]. One study included medical conditions such as asthmatic versus non-asthmatic participants [38].

The intensity and duration of smoking were well characterized, with several studies noting a minimum cumulative exposure. In [20,41], smokers had a median exposure of 18 pack-years, while [36] restricted smokers to ≥10 pack-years. The study also distinguished between former smokers (abstinent ≥ 12 months) and active smokers (smoked within 3 days). Refs. [39,43] specified minimum thresholds of ≥100 lifetime cigarettes. Both studies went a step further to describe daily use of cigarettes. Ref. [42] reported median smoking histories of 15 years and 15 cigarettes/day among current smokers. Ref. [44] extensively classified active smokers into long-term (≥10 cigarettes/day and ≥10 pack-years), short-term heavy (≥10/day and <10 pack-years), and mild (<10/day and <5 pack-years). A study analyzed an additional high-exposure subgroup with >10 pack-years (*n* = 159) [38]. Several studies also distinguished current from former smokers such as [36,38,44]. Some even defined e-cigarette users as daily/frequent users for ≥6 months with no use of combustible cigarettes for at least 6 months [39].

The exposure groups were generally made up of otherwise healthy adults. Exposed participants typically fell within adult age. For example, healthy smokers and/or EC users aged 18–50 or 18–40 years [34,35], young adults aged 21–30 years [39], and broad age adult samples such as 21–65 years [37] and 18–80 years [20]. Some studies focused on older or sex-specific populations, including adults > 40 years with substantial smoking histories [36], and a “Baby Boomer” cohort (born 1946–1964) [38]. Similar to the control groups, many studies excluded recent antibiotic use, acute respiratory illness, or concomitant illnesses. Across studies, exposed groups were drawn from bronchoscopy populations [39,41], dental and oral health clinics [42], and general community or volunteer groups [37,38], but were consistently described as clinically stable or free of acute respiratory infection at the time of sampling. The consensus from these data is that the study groups represent adult populations with well-defined smoking, e-cigarette, carriage, or respiratory disease exposure, with exposure intensity and duration quantified in most studies.

#### 3.1.5. Classification of Smoking-Related Exposures Across Included Studies

Since the included studies used heterogeneous definitions of smoking-related exposure, we grouped exposure definitions into broad categories instead of treating all exposures as equivalent. The included categories were current combustible cigarette smoking, former smoking, ENDS/e-cigarette use, and biochemically characterized active smoke exposure. Each type of exposure differs in combustion products, nicotine delivery, particulate matter, exposure intensity, duration, and route of exposure. Hence, findings were interpreted by exposure type and anatomical niche when possible. Given the substantial variation in exposure definitions, all exposure types were not assumed to have equivalent microbiome effects. The exposure categories are summarized in Table 1. Detailed study-level exposure definitions are provided in Supplementary Material S3, Table S1.

Only one study used biochemical markers to characterize active smoking exposure. Passive smoke/ETS exposure data from that study were not included in the core synthesis. In addition, secondhand smoke/ETS studies were not represented among the core adult respiratory microbiota studies. Therefore, ETS-related evidence is discussed separately as an adjacent exposure framework rather than pooled with active smoking or ENDS-related exposures.

No included study provided eligible respiratory microbiota data for waterpipe/hookah or heated tobacco product exposure. These exposure categories were considered in the search framework but were not analyzed as separate categories. Therefore, no cross-product comparison between waterpipe/hookah and the other exposure types should be inferred.

#### 3.1.6. Confounder Reporting and Handling Across Included Studies

Respiratory microbiota can be influenced by demographic, clinical, behavioral, and technical factors. Such factors may confound associations between smoking-related exposures and microbiota composition. Thus, we summarized how key potential confounders were reported or addressed across the core studies. These domains included age and sex, recent antibiotic exposure, or infection, respiratory or clinical disease status, oral health or periodontal status, and sequencing, sampling, or bioinformatic methodology. Detailed study-level characteristics and microbiome methods are provided in Supplementary Material S3, Tables S1 and S2. Table 2 summarizes the findings. “Reported” indicates that the variable was described in the study characteristics or methods. “Addressed by exclusions” indicates that the study excluded participants based on that factor such as recent antibiotics, recent infection, or clinical disease. “Adjusted” indicates that the factor was included in statistical adjustment. “Not clearly reported” indicates that the domain could not be confidently identified from the extracted study characteristics.

Overall, confounder reporting and handling varied across the included studies. Age, sex, clinical status, and sequencing methodology were commonly described whereas oral health/periodontal status and biochemical verification of smoking exposure were less consistently addressed. This variability supports cautious interpretation of smoking-associated microbiota shifts, particularly for oral/oropharyngeal findings that may be influenced by periodontal ecology and for low-biomass lower-airway samples that are sensitive to technical variation.

### 3.2. Smoking-Associated Changes in Respiratory Microbiota Diversity

We extracted and summarized alpha-diversity metrics (richness and diversity indices) from each of the 13 studies comparing smoking-related exposure groups with non-smoking, or never-smoking groups in upper and lower airways (Supplementary Material S3, Table S2). For the alpha-diversity findings, we performed a sub-group analysis of the results where data permitted. The core studies that reported findings were stratified by airway site (upper vs. lower airway) and specimen type (oral/oropharyngeal vs. BAL). The number of studies eligible for quantitative analysis were limited, therefore subgroup analyses were interpreted descriptively. Some studies contributed to more than one airway site synthesis (sampled more than one airway site).

#### 3.2.1. Alpha Diversity Changes Across Airway Sites (Upper vs. Lower Airway) and Specimen Type (Oral/Oropharyngeal vs. BAL)

##### Upper Airway vs. Lower Airway

In the 11 upper airway studies included in the alpha-diversity synthesis, Shannon or a similar Shannon metric was the most commonly used alpha-diversity measure (10/11 studies), followed by richness-based indices (8/11 studies) and Simpson-family metrics (6/11 studies). Phylogenetic diversity was less frequently reported (2/11 studies). In general, the results varied but a decline in alpha diversity among smokers was more frequently noted than an increase. A total of 5/11 studies indicated reduced diversity in smokers at various upper-airway locations, and 2/11 indicated increased diversity in smokers.

Only 3/11 studies observed no distinct smoking-related difference, and a single study did not provide alpha diversity results. The most noticeable trend of diminished diversity was observed in the oropharyngeal/pharyngeal and laryngeal areas. Paulo et al. (Hill q = 0, q = 1, and q = 2) [45], Bach et al. (Richness, Shannon, and Simpson) [34], and Turek et al. (Shannon) [38] noted decreased diversity among smokers in the oropharynx/pharynx. Jette et al. found reduced Shannon diversity in smokers within the larynx [37]. Contrarily, nasal and nasopharyngeal findings were less consistent. Hickman et al. and Yu et al. found no distinct variations at nasal locations [35,42], and Paulo et al. noted only slight differences in the nasopharynx [45]. Charlson et al. indicated greater diversity among smokers in both the nasopharynx and oropharynx [43]. Oral findings were site dependent. Ying et al. documented no differences [39], Morris et al. found no evident impact of smoking [20], and Yu et al. reported that most oral sites remained unchanged, except buccal mucosa (lower diversity in smokers) [42]. Gioula et al. likewise showed no notable smoking-associated variations in pharyngeal richness or Shannon diversity [40].

In the lower airway studies (five studies), Simpson-family metrics were the predominant alpha-diversity measure used (4/5 studies). It was followed by Shannon or a Shannon-equivalent metric (3/5 studies), and richness-based indices like Chao1 or observed OTUs/species (3/5 studies). Only 2/5 studies used phylogenetic diversity. Unlike the upper airway, the findings were inconsistent and showed limited evidence for a stable smoking-associated alpha diversity pattern. Campos et al. indicated reduced diversity in smokers, with lower Chao1 richness, inverse Simpson diversity, and Faith’s phylogenetic diversity [36]. Ying et al. and Liu et al. showed no significant smoking-related differences (2/5 studies) [39,41] and Pfeiffer et al. did not report alpha diversity findings [44].

##### Oral/Oropharyngeal vs. BAL

In the upper airway studies, oropharyngeal swabs were the most frequently used specimen (five studies). They also showed the clearest tendency toward reduced alpha diversity in smokers. Paulo et al. (Hill q = 1 and q = 2), Bach et al. (richness, Shannon, and Simpson), and Turek et al. (Shannon), all reported lower diversity in smokers [34,38,45]. Charlson et al., using Shannon and Chao1, reported higher diversity [43]. On the other hand, nasal-type specimens were largely null. One study that used Shannon and Simpson in nasal epithelial lining fluid/nasal lavage fluid, found no overall inter-group difference [35]. Another study also reported that there is no smoking-associated difference in diversity in the anterior nares using observed species, Shannon, and phylogenetic diversity [42]. Nasopharyngeal swabs showed variability. Paulo et al. noted only marginal differences across Hill q = 0, q = 1, and q = 2 [45], whereas Charlson et al. observed greater Shannon diversity and Chao1 richness among smokers [43]. Oral specimens were more heterogeneous. Saliva-based studies showed no clear smoking-related difference [39,42]. Morris et al. used observed OTUs, Shannon, inverse Simpson, and phylogenetic diversity, and found no effect in the V1–V3 analysis in oral washes [20]. One oral study found that most oral subsites were unchanged except for the buccal mucosa, where smokers had lower observed species and phylogenetic diversity [42]. It also reported a near-significant reduction in Shannon diversity [42]. A pharyngeal study sampled pharyngeal swabs and found no significant difference using richness and Shannon indices [40].

When the lower airway studies were stratified by specimen type, BAL was the most frequently used sample (4/5 studies). However, it also showed the greatest heterogeneity in alpha-diversity findings. Only one study among these four reported lower diversities in smokers [36]. One study showed no changes in observed species, Shannon, and Gini-Simpson [39]. Morris et al. noted inconsistent richness findings across sequencing regions. They found no smoking-related effect in the V1–V3 analysis, and only a V3–V5 richness signal that was not consistent across BAL and oral washes [20]. One study did not report any findings [44]. A BALF study that used Shannon and Simpson found no significant difference between smokers and non-smokers.

This subgroup analysis shows that oropharyngeal swab literature mainly contributes to the pattern of reduced alpha diversity in smokers. Nasal, nasopharyngeal, and oral specimen types show more variability. The lower airway analysis suggests that any smoking-related alpha diversity signal is driven mainly by the BAL literature and remains inconsistent even there.

#### 3.2.2. Beta Diversity and Community Separation by Smoking Status

Smoking was variably associated with differences in beta-diversity in the lower-airways (Supplementary Material S3, Table S2). Ref. [36] reported significant Bray–Curtis dissimilarity between never, former, and current smokers in BAL, with smoking explaining a measurable fraction of variance in PERMANOVA and visible separation of groups in ordination spaces [36]. Similarly, a larger cohort identified that current smoking was a major covariate influencing airway community structure in multivariable models, revealing notable variations in beta-diversity between smokers and non-smokers even after controlling for asthma and cumulative exposure [38]. By contrast, other lower-airway data have all reported significant overlap of groups and non-significant PERMANOVA results, with beta-diversity affected by disease status or inter-individual variation rather than smoking alone [20,39,41].

Similarly, in the upper airways (Supplementary Material S3, Table S2), there were heterogeneous beta-diversity patterns, but smoking signals were more reproducible in the oropharynx and combined naso/oropharyngeal sites. Initial research using 454 sequencing demonstrated that the nasopharyngeal and oropharyngeal microbiotas of smokers constituted a distinct cluster and exhibited more disorder than those of non-smokers as indicated by UniFrac-based ordination [43]. More recent 16S rRNA V4 sequencing studies in adult pneumococcal carriers likewise identified smoking as a modest predictor of nasopharyngeal and oropharyngeal community composition in PERMANOVA models [45]. Longitudinal pharyngeal sampling showed consistent Bray–Curtis/UniFrac separation between smokers and non-smokers, with further divergence during antibiotic exposure and viral colds [34]. Oral wash also showed clear compositional differences between smokers and non-smokers despite similar alpha-diversity [20]. In contrast, large multi-site oral/nasal studies found that beta-diversity was determined by anatomical site and individual identity, with smoking contributing minimally to overall inter-sample variation and no strong clustering by status was found at most oral or nasal sites [40,42]. Nasal NELF studies similarly reported only modest exposure-related beta-diversity differences among smokers, e-cigarette users, and non-smokers [35].

These findings indicate that smoking can be detected as a beta-diversity driver in both upper and lower airways, but as a minor effect overshadowed by strong site and host-driven structure. Smoking status frequently appears as a statistically significant predictor of community composition in situations where contrasts in exposure are high and cohorts are specific, such as never versus long-term smokers in BAL or well stratified oropharyngeal and nasopharyngeal cohorts [34,36,38,45]. However, in many studies, beta-diversity differences between smokers and non-smokers are small or absent, particularly in nasal and some oral datasets [35,39,42], understanding that smoking-related restructuring of the respiratory microbiota is context dependent.

### 3.3. Site-Specific Taxonomic Shifts Associated with Smoking Across the Airway Axis

**Figure 2:**
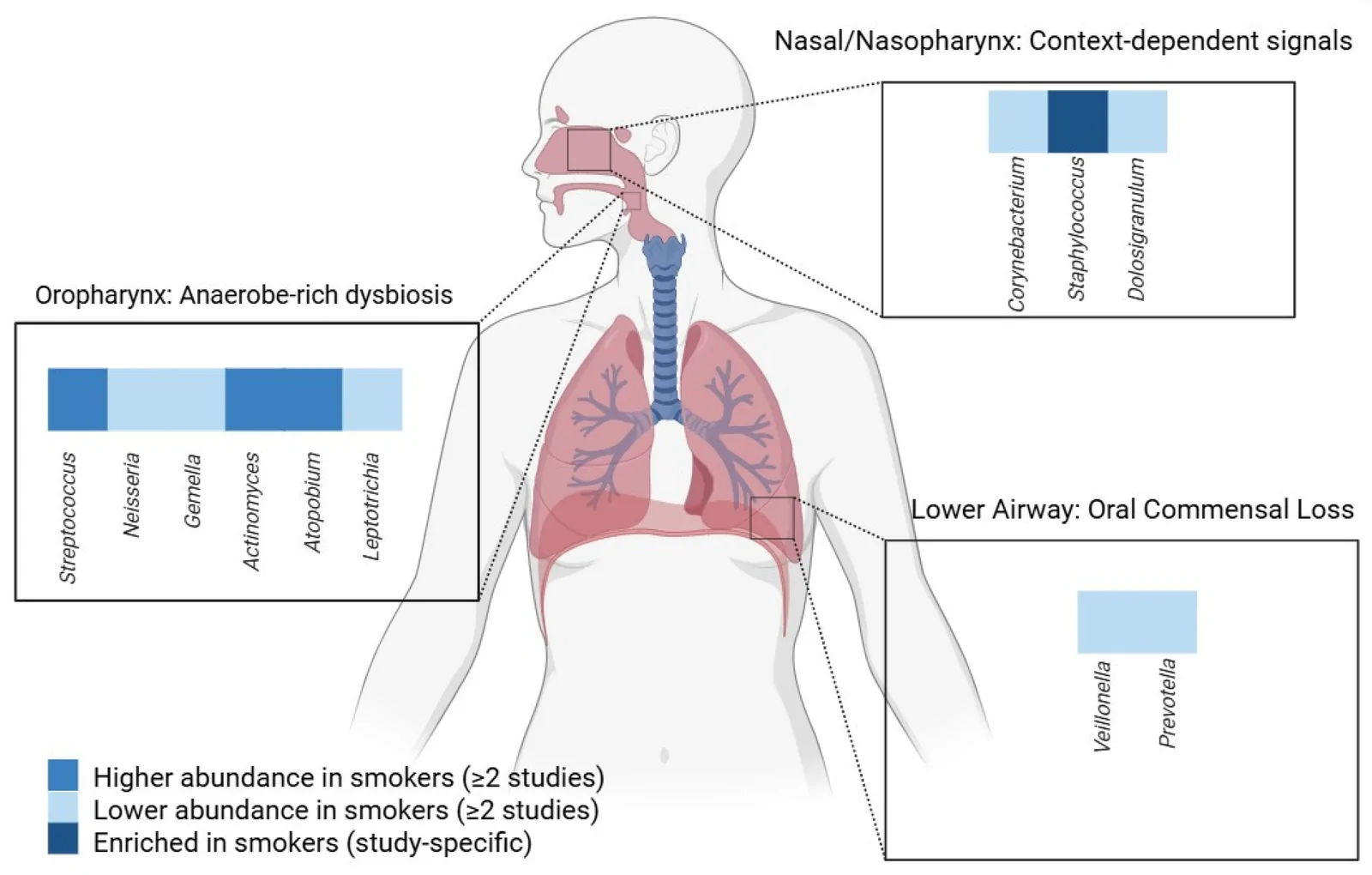
Site-stratified heatmap of smoking-associated genera across the airway axis. The heatmap is intended as a narrative direction-of-effect summary. Recurring signals indicate taxa reported in the same direction in ≥2 studies within the same airway site. The schematic shows important genera that were found to be at higher or lower abundance in smokers compared to non-smokers across the core studies in three respiratory niches: nasal/nasopharynx, oropharynx, and lower airway. Direction and consistency are indicated by color: dark blue = study-specific enrichment in smokers; light blue = lower abundance in smokers (≥2 studies); and medium blue = higher abundance in smokers (≥2 studies). An increase in *Streptococcus*, *Actinomyces*, and *Atopobium* and a decrease in *Neisseria*, *Gemella*, and *Leptotrichia* denotes an anaerobe-rich dysbiosis pattern in the oropharynx. The lower airway shows consistent depletion of *Veillonella* and *Prevotella*. Nasal/nasopharyngeal signals have shown to be more context-dependent and highlighted a depletion of *Corynebacterium* and *Dolosigranulum* with study-specific enrichment of *Staphylococcus*. This is an original figure created for this review.

Across 13 studies sampling upper- and lower-airway sites (nasal/nasopharynx, oropharynx, and lower airways), we summarized taxa reported as having higher or lower abundance in smokers vs. non-smokers at the phylum, genus and species levels and visualized the recurring signals (reported in ≥2 independent studies) as site-stratified heatmaps (Supplementary Material S4). Figure 2 illustrates our key findings.

Taxa were included in the heatmap when the original studies reported a smoking-associated difference in relative abundance or differential abundance within a given airway niche. The heatmap was intended as a narrative direction-of-effect summary. Statistically significant findings were prioritized when available. Explicitly reported directional taxa-level differences were also included when significance was not comparable or not available. Adjusted and unadjusted results were not pooled or treated as equivalent effect estimates. Consistent signals were defined as taxa reported in the same direction in at least two studies within the same broad airway site. Contradictory findings were retained but were not interpreted as consistent signals unless the same direction was observed in at least two studies. Genus-level taxa were manually harmonized by standardizing taxon names. On the other hand, species-level findings were summarized separately because 16S-based species assignments vary across sequencing regions, reference databases and bioinformatic pipelines. Hence, the findings at this taxonomic level were interpreted more cautiously given the varying confidence in taxonomic identification.

#### 3.3.1. Upper Respiratory Tract

A total of 11/13 studies investigated the effects of smoking on the upper airways. Specifically, these studies investigated the oral cavity, oropharynx, nasopharynx, and larynx. In the oral/oropharynx cohorts (eight studies), only two reported phylum-level smoking associations. The most consistent finding was higher abundance of Actinobacteria in the oropharynx of smokers [34,44]. Ref. [44] additionally noted an increased abundance of Firmicutes in smokers and reductions in beta-proteobacteria. None of the studies highlighted phylum-level findings in the nasal/nasopharynx/larynx cohort (Supplementary Material S4). At the genus level, the most consistent oropharyngeal smoking signature emerged across seven cohorts (Supplementary Material S4) [20,34,38,39,43,44,45]. Specifically, a recurrent higher abundance of *Streptococcus* (three studies), *Actinomyces* (three studies), *Veillonella* (two studies) and *Atopobium* (two studies) were reported in smokers [34,38,39,43,44]. There was a consistently reported lower abundance of *Neisseria* (five studies), *Haemophilus* (two studies), *Fusobacterium* (two studies), *Gemella* (two studies), *Leptotrichia* (two studies) and *Capnocytophaga* (two studies) (Supplementary Material S4). In nasal/nasopharyngeal/laryngeal samples, six studies reported findings [35,37,40,43,44,45]. However, smoking-associated genus-level signals were subtler and more variable. One study noted the depletion of *Corynebacterium* and *Dolosigranulum* [44] with species-level results in one study supporting this pattern [45] (Supplementary Material S4). Another study reported an increase in abundance in *Bifidobacterium*, *Alloscardovia*, *Dialister*, and *Filifactor* but a decrease in *Moraxella* and *Gemella* in smokers [40]. Due to the large variability in the results, species-level data were reported in Table S4 in Supplementary Material S8. Species-level results were reported less consistently and were less comparable across studies. Nasal species findings were largely study-specific (Supplementary Material S8, Table S4). One e-cigarette study reported enrichment of *Staphylococcus aureus* in e-cigarette smokers compared to cigarette smokers and non-smokers [35]. Another study reported the enrichment of *Rothia dentocariosa*, *Prevotella melaninogenica*, and *Veillonella atypica* in smokers [45].

#### 3.3.2. Lower Respiratory Tract

In the lower airway (five studies), smoking was most consistently associated with lower Bacteroidetes and Firmicutes abundances in smokers [36,41]. A Proteobacteria shift was highlighted, where higher Proteobacteria was reported in two studies [36,41] (Supplementary Material S4). In lower-airway cohorts (three studies), smokers most consistently showed reductions in the abundance of *Veillonella* [36,41,44] and *Prevotella* [36,41] (Supplementary Material S4). There was a mixed direction reported for *Streptococcus*, where one study [41] reported an increase whereas the other noted a decrease in abundance in smokers [36]. The remainder of the findings across the studies were heterogeneous. Like the upper airway species findings, the lower airway exhibited variability in the results. Only two lower-airway studies reported species-level shifts (Supplementary Material S8, Table S4) [39,44]. Ref. [39] reported increases in *Niastella koreensis*, *Myroides odoratimimus*, *Lactobacillus kefiranofaciens*, among others. Ref. [44] reported an enrichment of gamma-Proteobacteria species, specifically, *Acinetobacter bereziniae*, *A. johnsonii*, *Cupriavidus metallidurans*, *Serratia marcescens* and *Stenotrophomonas maltophilia*. Ref. [39] noted diminished abundance of *Neisseria elongata*, *Neisseria sicca*, and *Haemophilus parainfluenzae* in smokers, whereas ref. [44] reported lower abundances in *Prevotella* and *Veillonella* species.

Our findings show that smoking is associated with distinct but variable alterations in the respiratory microbiota. The clearest genus-level shifts were seen in the oropharynx. At this respiratory niche, smokers showed higher abundances of *Streptococcus*, *Actinomyces*, *Veillonella*, and *Atopobium. Neisseria* showed the most consistent lower abundance signal in oropharyngeal cohorts. *Haemophilus*, *Fusobacterium*, *Gemella*, *Leptotrichia*, and *Capnocytophaga* had lower abundances in smoker’s oropharynx. In the lower airway, a lower abundance of *Veillonella* and *Prevotella* was the strongest signal while other lower airway findings were more heterogeneous. These results support a smoking-related, site-specific microbial signature across the respiratory tract.

### 3.4. Exploratory Meta-Analysis of BAL Inverse Simpson Diversity in Smokers vs. Non-Smokers

An inverse-variance random-effects model meta-analysis was conducted using standardized mean differences (SMDs) (Hedges g). Two studies contributed to this analysis: [20,36]. Both were observational, cross-sectional studies and were assessed to have low to moderate risk of bias based on the JBI checklist. Ref. [36] reported substantially lower diversity in smokers compared with never-smokers (SMD −3.42, 95% CI −4.62 to −2.23), whereas ref. [20] observed no meaningful difference between smokers and non-smokers (SMD 0.09, 95% CI −0.54 to 0.72). The pooled estimate from the random-effects model (REML heterogeneity estimator, Wald-type CIs) was an SMD of −1.63 (95% CI −5.07 to 1.81), and the test for the overall effect was not statistically significant (Z = 0.93, *p* = 0.35).

**Figure 3:**
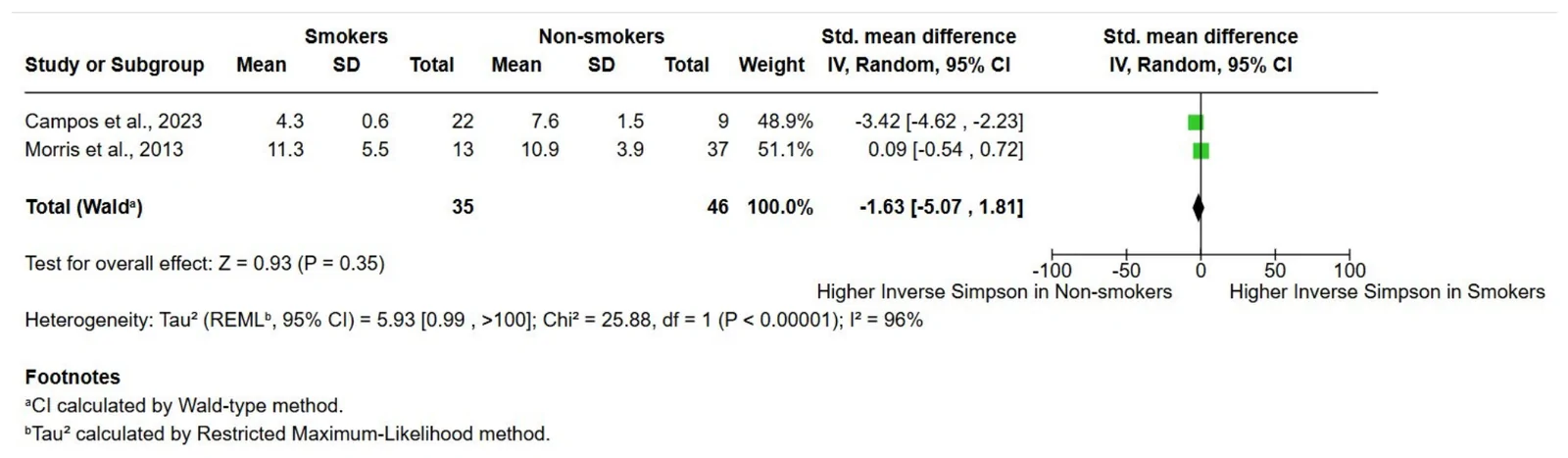
Forest plot of the exploratory meta-analysis of BAL inverse Simpson diversity in smokers vs. non-smokers. For bronchoalveolar lavage (BAL) inverse Simpson diversity, individual study effects are displayed as standardized mean differences (Hedges g) between smokers and non-/never-smokers; square size represents study weight, horizontal lines represent 95% CIs, the diamond represents the pooled random-effects estimate. An overall SMD of −1.63 (95% CI −5.07 to 1.81; *p* = 0.35) was obtained by pooling estimates from the two eligible studies Morris et al. [20] and Campos et al. [36] using a random-effects model (REML heterogeneity estimator; Wald-type CIs). The pooled estimate should be interpreted with caution because the heterogeneity was extreme (I^2^ = 96%, χ^2^ = 25.88, df = 1, *p* < 0.00001), indicating significant inconsistency between studies. This pooled estimate is presented as an exploratory, hypothesis-generating summary rather than a definitive quantitative conclusion.

Inter-study heterogeneity was extremely high (I^2^ = 96%, χ^2^ = 25.88, df = 1, *p* < 0.00001), indicating that the effect sizes from [20,36] are highly inconsistent and unlikely to reflect a single underlying effect. Accordingly, this two study BAL meta-analysis should be considered exploratory and hypothesis-generating only. It should not be used to infer the magnitude of the association between smoking status and BAL inverse Simpson diversity. Overall, the available evidence does not provide a consistent or precise estimate of the association between smoking status and BAL inverse Simpson diversity, and the direction and magnitude of any true effect remain uncertain. The forest plot can be seen in Figure 3.

## 4. Discussion

### 4.1. Biological Interpretation of Site-Specific Microbiota Signatures Along the Airway Axis

#### 4.1.1. Oropharynx: Anaerobe-Enriched Dysbiosis Consistent with Inflammation and Low Oxygen Microenvironments

According to the studies included in this review, smokers showed a consistent pattern in the oropharyngeal microbiota with higher levels of anaerobic Firmicutes and Actinobacteria. These included genera such as *Streptococcus*, *Actinomyces* and *Atopobium*. At the same time, smokers showed a *reduction* in common Proteobacteria and Fusobacteria commensals including *Neisseria*, *Haemophilus*, *Fusobacterium*, *Leptotrichia*, and *Gemella*. This microbial pattern is compatible with an inflamed mucosal environment, as cigarette smoke contains reactive substances that damage epithelial cells and increase local inflammation [46]. Inflammatory conditions tend to favor the growth of anaerobic genera such as *Actinomyces*, while more sensitive commensals such as *Neisseria* decline [20]. Moreover, studies have shown that inflamed airways select for the proliferation of biofilm forming taxa [47,48,49], such as *Actinomyces* and *Atopobium*. Ref. [43] showed that smokers had more irregular and less stable Naso-oropharyngeal communities than non-smokers, which supports this interpretation.

The shift observed in smokers also suggests lower oxygen availability in the oropharynx. Smoke exposure alters blood flow and oxygen delivery in the oral tissues, which results in a more anaerobic environment [50]. Under low-oxygen conditions, facultative and obligate anaerobes including *Streptococcus* increase in abundance, while aerobic Proteobacteria decrease [44].

The composition in smokers also resembles the microbial profile commonly seen in periodontal disease. Several taxa enriched in smokers, including *Actinomyces* and *Atopobium* are recognized components of dysbiotic subgingival biofilms [50]. Smoking is a strong risk factor for periodontal disease and is known to promote the growth of anaerobic complexes while reducing beneficial commensals [51]. Ref. [52] also reported that clinically healthy smokers had a subgingival microbial profile that shifted away from health-associated communities toward more dysbiotic, disease-associated assemblages [52].

The consistent anaerobe-enriched oropharyngeal profile in smokers is clinically relevant because the oropharynx is a major source community for micro-aspiration into the lower airways, especially in older adults, patients with chronic lung disease, and perioperative populations [53]. If smoking is associated with anaerobe-rich dysbiosis and reduced protective commensals, this pattern could plausibly be linked to higher risk of adverse respiratory outcomes like COPD [54], although the available studies do not establish causality. These findings also support the view that oral health is a modifiable co-factor in smoke-related respiratory risk. Future studies should measure periodontal status and test whether periodontal treatment and/or smoking cessation shifts the oropharyngeal microbiome toward a more “health-associated” composition and whether that correlates with improved respiratory outcomes [51,52].

#### 4.1.2. Heterogeneous Changes and Context Dependence Across Nasal/Nasopharyngeal/Laryngeal Cohorts

In the nasal cavity and nasopharynx, the microbial response to smoking appears heterogeneous. While phylum-level alterations were absent, genus-level analyses reveal subtle shifts. One study’s findings hint at a potential disruption of the local microbial homeostasis by describing a reduction in commensal genera associated with respiratory health (*Corynebacterium*, *Dolosigranulum*). This finding was confirmed by species-level data in another study which reported reductions in *Corynebacterium propinquum*/*C. pseudodiphtheriticum* and *Dolosigranulum pigrum*/*uncultured Alloiococcus* spp. *(ASV72)* [45]. *Dolosigranulum* spp. is a typical resident in the healthy nasopharynx [55]. Abundance of *Dolosigranulum pigrum* and *Corynebacterium* has actually been shown to be correlated with lower prevalence of URT disease states [56]. Experimental work has shown that *D. pigrum* can inhibit the growth of *Staphylococcus aureus* in vitro [57], suggesting a potential role in colonization resistance against *Staphylococcus.* A possible implication is that reduced colonization resistance could favor *Staphylococcus aureus* expansion in some contexts, but this remains speculative. It has been noted that cigarette smoke increases *S. aureus* biofilm production [49] which could make it more virulent. One study reported enrichment in *Bacillus* and *Burkholderia*, two taxa that were reported as being part of the cigarette metagenome, thus their source in the nasopharynx could be the cigarettes themselves [58].

The nasopharyngeal microbiome is strongly shaped by host factors such as age and by environmental exposures, including seasonal variation, leading to substantial inter-individual variability [59]. Prior large-scale analyses similarly show that URT microbial patterns differ across geography and sampling site, underscoring that differences observed at this niche are highly context-dependent and often influenced by study design and methodology [60]. Moreover, the subtle smoking-associated signal may also reflect general methodological limitations of 16S-based respiratory microbiome studies, including differences in sampling, sequencing, and bioinformatic analysis, which can introduce variability and obscure modest community-level shifts [61].

Clinically, the heterogeneity across nasal/nasopharyngeal studies suggests that this niche may be a weak standalone biomarker of smoking exposure without careful control of confounders (seasonality, age, geography, sampling depth). However, the depletion of commensals associated with upper-airway stability (e.g., *Corynebacterium*/*Dolosigranulum*) raises the hypothesis that smoking could reduce colonization resistance, potentially altering pathogen carriage dynamics [62]. Prospective studies that link nasal microbiome changes to incident upper respiratory infections, carriage status, symptom burden, and immune markers would clarify whether these subtle shifts have meaningful clinical predictive value.

#### 4.1.3. Passive Smoke/ETS Exposure and Early Life Upper-Airway Microbiota

Although the core synthesis focused mainly on adult active smoking and related products, the upper-airway findings are also relevant to passive smoke/ETS exposure because nasal and oral microbial communities may be affected during early-life exposure windows. Unlike active smoking in adults, ETS exposure differs in dose and route of exposure. Therefore, its microbiome effects should not be assumed to be equivalent to those of active smoking. Nevertheless, emerging evidence suggests that ETS may influence microbial communities in airway-adjacent niches. In a pilot multi-omic study of 56 children, Zhang et al. reported that ETS exposure during pregnancy and early childhood was associated with overall nasal and oral microbiota composition [63]. They also found that early-childhood ETS exposure was associated with changes in oral microorganisms such as enrichment of potentially risky taxa [63]. This suggests that smoking-related exposure may alter upper-airway microbial composition even in individuals who are not active smokers. Since early-life ETS studies were not represented among the core adult studies in this review, this analysis should be interpreted as complementary to the pooled active-smoking evidence.

#### 4.1.4. Lower Airway Remodeling in Smokers: Loss of Anaerobic Commensals and Opportunistic/Environment-Associated Proteobacteria Enrichment

Smokers showed lower abundances of *Veillonella* and *Prevotella* in the lower airways. Ref. [11] states that in healthy sputum samples, the dominant taxa are Firmicutes/Bacteroidetes with oral commensals like *Veillonella*, and *Prevotella*, which were notably reduced in our results. These genera likely entered the lower airways through ongoing micro-aspiration from the oropharynx. Experimental and translational studies suggest that these taxa are not merely bystanders but can participate in airway immune regulation. A study showed that episodic aspiration of species under these genera induced a MyD88-dependent pulmonary Th17 response and reduced susceptibility to *Streptococcus pneumoniae* in mice [64]. Another study reported that airway *Prevotella* has also been shown to promote neutrophil activation and more rapid pneumococcal clearance [65]. One plausible explanation is that cigarette smoke may alter the airway niche via oxidative stress [66] and pro-inflammatory responses [67]. These mechanisms may make the lower airway less hospitable to anaerobe-associated commensals such as *Prevotella* and *Veillonella.* This interpretation is strengthened by overlap with respiratory disease-associated dysbiosis. In COPD, increasing severity of disease has been associated with reduced *Prevotella* [68]. In the microbiota of patients with severe and non-severe asthma, *Veillonella* and *Prevotella* were shown to be less common [69]. Collectively, this information suggests that the lower abundance of *Prevotella* and *Veillonella* in the lower airways of smokers may represent an early smoking-related shift from a commensal lower airway microbiota toward a profile that overlaps with chronic respiratory disease such as COPD and asthma.

Moreover, our synthesis has found that non-fermenting/environmental Proteobacteria species (*Acinetobacter*, *Cupriavidus*, *Serratia*, *Stenotrophomonas*) were found in the lower airways. Studies in COPD, a prototypical smoke-related lung disease, similarly describe low-diversity communities dominated by Proteobacteria that are intricately linked to neutrophilic inflammation [70,71]. Both studies support the idea that smoke-related lower-airway disease is characterized by loss of diverse oral commensals and overgrowth of stress-tolerant, potentially pathogenic Proteobacteria. *Ralstonia*, *Burkholderia*, *Acinetobacter*, *Stenotrophomonas*, *Cupriavidus*, and *Serratia* are all environmental/water-associated, opportunistic pathogens ([72,73,74,75,76]). These gamma-proteobacteria species are capable of biofilm formation. For instance, *Stenotrophomonas maltophilia* has the capacity to form biofilms and can produce antibiotic-inhibiting beta-lactamases [77]. Some of these Proteobacteria, like *Serratia marcescens*, can be transferred to the lungs via tobacco flakes since they can grow on cigarettes [78]. Together, this pattern is consistent with the possibility that smoking-associated airway conditions may be less hospitable to commensal taxa. Importantly, because distal airway specimens are low biomass, “environmental/water-associated” taxa can reflect either true airway colonization or technical contamination [79]. Therefore, these sequencing signatures should not be interpreted as infection requiring treatment without supportive clinical microbiology.

Across the airway axis, smoking is associated with site-dependent microbiota remodeling rather than a uniform compositional shift. In the oropharynx, the most consistent signal is an anaerobe-enriched dysbiosis that overlaps with periodontal-type community configurations and could plausibly be associated with lower respiratory tract infection risk via oral–lung microbial seeding. In the nasal/nasopharyngeal/laryngeal niche, smoking-related changes are subtle and highly context-dependent, suggesting erosion of “health-associated” commensals. Strict confounder control may help determine whether this niche has biomarker potential for smoking-associated microbial shifts. In the lower airways, loss of anaerobic commensals resembled microbial patterns found in chronic respiratory diseases such as COPD and asthma. In addition, the reported loss of oral commensals with enrichment of opportunistic/non-fermenting Proteobacteria is a pattern concordant with smoke-related chronic airway disease and the presence of potential pathobionts. Due to the low biomass and transient nature of the lower-airway micro-niche, cautious interpretation of this finding is needed.

### 4.2. Interpreting Inter-Study Heterogeneity and Sub-Group Analyses

The core studies varied substantially in design, populations, sampling strategy, sequencing methods, and exposure definitions. This variability was especially evident in the two-study BAL meta-analysis, where heterogeneity was extreme (I^2^ = 96%, τ^2^ = 5.93). This indicates that the pooled estimate should be viewed as exploratory rather than as a robust estimate of the true association. The two BAL studies differed in participant populations, underlying respiratory diagnoses, and exclusion criteria for recent infection, antibiotic exposure, and inhaled corticosteroid use [20,36]. More broadly, across the core 13 studies there was considerable variation in sampling strategy. Some studies collected oral wash, nasal swabs, nasopharyngeal aspirates, and BAL, each targeting distinct airway compartments with different baseline communities and biomass levels. DNA extraction protocols, 16S rRNA regions that were sequenced, and sequencing platforms (454 vs. Illumina) also varied between studies. Moreover, species-level signals were sometimes reported as ASVs rather than resolved species, such as in [45], limiting cross-study comparability. Also, several significant ‘enriched’ species were low-prevalence or environmentally associated taxa, such as *Niastella* and *Polaribacter* in [39], suggesting that species-level calls in low-biomass airway samples may be sensitive to pipeline/contamination effects. The definition of non-smokers and smokers varied across the studies as shown in the Section 3.1.3 and Section 3.1.4. Several studies excluded respiratory conditions such as asthma and COPD whereas others deliberately enrolled COPD patients or asthmatics or even included e-cigarette users as a separate group. Differences in clinical context, age, and sex distributions were not uniformly controlled across studies, which further limits comparability.

There was a difference in the alpha diversity metrics used in each study as shown in the earlier subgroup analyses. The subgroup analyses were performed to contextualize the meta-analysis and to assess whether additional studies were sufficiently comparable for quantitative synthesis. Our analyses showed substantial heterogeneity across the included studies in terms of airway niche, specimen type, alpha-diversity metric used, and manner of reporting the data. Taken together, these subgroup findings support the restriction of the meta-analysis to the two studies with sufficiently comparable BAL inverse Simpson data. Thus, it suggests that the limited quantitative synthesis reflects the heterogeneity of the available literature. The subgroup analyses strengthen the meta-analysis by demonstrating why broader pooling was not methodologically justified.

### 4.3. Strengths and Limitations

This review has several strengths. It used a predefined protocol, a comprehensive multi-database search strategy, and explicit inclusion and exclusion criteria to identify observational studies examining smoking and the respiratory microbiota across a range of respiratory niches. Data extraction and synthesis were structured to allow comparison of alpha and beta diversity, taxonomic shifts, and, where possible, quantitative pooling of diversity measures. By systematically separating upper and lower airway sites and considering different smoking exposures, the review provides a nuanced overview of how and where smoking may influence respiratory microbial communities.

This systematic review lacked prospective registration, which may have increased the risk of reporting bias and deviation from protocol. The conclusions of this review are constrained. Only a small number of studies, notably just two, were suitable for meta-analysis, limiting the ability to derive precise pooled estimates. This restriction was due to the methodological variations across many studies for the alpha diversity metric. This made it difficult to include more studies in the meta-analysis. The heterogeneity in study design, populations, sampling, and analytical methods restricted the possibility of quantitative synthesis for most outcomes. Restriction to databases and the likelihood of publication bias mean that some relevant studies may not have been captured. Study quality and risk of bias also limit the strength of our conclusions. Across the 13 core articles, methodological quality assessed using the MINORS tool was generally moderate rather than high, with a mean score of 14.9 ± 1.2 (range 14–18). These mid-range scores indicate that most studies met several basic methodological criteria but commonly failed or only partially met others, such as prospective sample size calculation, clear reporting of consecutive or representative inclusion, and explicit use of blinded outcome assessment. Many cohorts were small, and cross-sectional, which limits statistical power and makes effect estimates vulnerable to random error and residual confounding variables. MINORS and JBI provided structured assessment of observational study quality; however, they do not fully capture microbiome-specific technical sources of bias. The microbiome-specific methodological assessment showed heterogeneous reporting of contamination controls, contaminant filtering, read-depth thresholds, and low-biomass handling, which supports the cautious interpretation of lower-airway findings, particularly for BAL/BALF studies. All these factors are likely to influence both diversity measures and compositional shifts. Species-level taxonomic findings should be interpreted with caution, especially when inferred from 16S rRNA sequencing or reported in low-biomass samples. Species-level resolution is more sensitive to sequencing region, reference database, bioinformatic pipeline, and contamination-control procedures than genus-level classification. These issues mean that the overall confidence in the evidence is limited by methodological and clinical heterogeneity, and the results of our meta-analysis and narrative synthesis should be interpreted in that context. Another limitation is that smoking and e-cigarette exposure status was often self-reported and not based on biochemical verification such as cotinine or exhaled carbon monoxide testing. This may have introduced exposure misclassification which could have attenuated the observed associations between smoking-related exposures and the respiratory microbiota. Moreover, exposure heterogeneity was treated as an important limitation of cross-study comparison. Thus, the current evidence does not support meaningful comparisons across different exposure types.

## 5. Conclusions

This systematic review suggests that smoking is associated with site-specific restructuring of the respiratory microbiota, most consistently in the oropharynx. In this site, smokers more often exhibit anaerobe-enriched communities, specifically in the Actinobacteria phylum. The strongest smoking-related signal in the oropharynx was a loss of *Neisseria*. Nasal/nasopharyngeal changes are generally subtler and more heterogeneous, whereas lower-airway samples more often shift away from oral commensals with loss of *Veillonella* and *Prevotella*. One lower-airway study highlighted enrichment of opportunistic/environment-associated taxa in the lower airways. However, this finding must be interpreted with caution considering low-biomass sampling and contamination risk. The distinct, site-specific dysbiosis patterns identified here may reflect smoking-associated remodeling of respiratory microbial communities and could plausibly participate in inflammatory or infection-related pathways. However, because most included studies were observational and cross-sectional, the available evidence supports association rather than causation.

Alpha-diversity results were mixed, with a preponderance of reduced diversity rather than a single reproducible increase outcome. This pattern was driven mainly by upper airway studies and was most often observed with Shannon/Simpson-type diversity metrics. Richness findings were more mixed, and lower airway studies were largely null or inconsistent. Our exploratory meta-analysis of lower-airway diversity was inconclusive because it included only two BAL studies and showed extreme between-study variability. These findings should therefore be viewed as a descriptive quantitative summary rather than a definitive estimate of effect. Beta-diversity analyses indicate that smoking can act as a driver of community composition in several cohorts, but it typically explains only a modest proportion of variance compared to inter-individual differences, sampling site and other host or environmental factors. In general, the available evidence suggests that smoking appears to reshape respiratory microbial networks along anatomical and ecological gradients. However, these conclusions are constrained by substantial methodological and clinical heterogeneity. Future studies should incorporate standardized multi-site sampling, and include adequately powered comparisons of combustible cigarettes, ENDS/e-cigarettes, waterpipe/hookah, and other tobacco products. Prospective studies should make use of metagenomics and metatranscriptomics and integrate host immune profiling. Such studies will be essential to determine whether smoking-related dysbiosis is primarily a marker of exposure or plays a biologically active role in respiratory infection susceptibility and chronic airway disease.

## Figures and Tables

**Table 1:** Summary of smoking-related exposure categories across included studies.

Exposure Category	Studies	Definition Examples	References
Current combustible cigarette smoking	Bach et al., Hickman et al., Campos et al., Jette et al., Morris et al., Turek et al., Ying et al., Gioula et al., Liu et al., Yu et al., Charlson et al., Pfeiffer et al., Paulo et al.	Current smoker status, cigarettes/day thresholds, lifetime cigarette exposure, recent smoking, or pack-year history.	[20,34,35,36,37,38,39,40,41,42,43,44,45]
Former smoking	Campos et al., Turek et al., Pfeiffer et al.	Former or ex-smoker status; abstinence period reported in some studies.	[36,38,44]
ENDS/e-cigarette use	Hickman et al., Ying et al.	E-cigarette users analyzed as a separate exposure group; frequency or duration criteria reported in some studies.	[35,39]
Biochemically characterized active smoke exposure	Pfeiffer et al.	Active smoking exposure classification using urinary nicotine, cotinine, 3-OH-cotinine, and anabasine.	[44]
Never-smoker/non-smoker comparison groups	All included studies	Non-smoker, nonsmoker, or never-smoker groups; lifetime cigarette thresholds such as <100 cigarettes were used in some studies.	[20,34,35,36,37,38,39,40,41,42,43,44,45]

**Table 2:** Study-level reporting and handling of key confounder domains across included studies.

Study	Age/Sex	Recent Antibiotics/Infection	Clinical Status	Oral/Periodontal Status	Sampling/Sequencing
Hickman	Reported	Addressed by exclusions	Addressed by exclusions	Not Reported	Reported
Pfeiffer	Reported	Not clearly reported	Healthy adults reported	Not reported	Reported
Paulo	Adjusted for age/gender	Recent antibiotic exclusion	Healthy adults reported	Not reported	Reported
Bach	Reported	Not clearly reported	Healthy adults reported	Not reported	Reported
Campos	Reported	Not clearly reported	Clinical groups reported	Not reported	Reported
Liu	Reported	Not clearly reported	Bronchoscopy population reported	Not reported	Reported
Gioula	Reported	Addressed by exclusions	Healthy volunteers reported	Not reported	Reported
Jette	Reported	Addressed by exclusions	Reflux strata and clinical exclusions reported	Not reported	Reported
Ying	Reported/balanced	Not clearly reported	Healthy adults; bronchoscopy	Not reported	Reported
Morris	Reported	Addressed by exclusions	Pulmonary disease excluded	Not reported	Reported
Yu	Reported/matches	Addressed by exclusions	Cancer excluded	Periodontal disease/dental cleaning excluded	Reported
Charlson	Reported	Addressed by exclusions	Healthy/asymptomatic adults reported	Not reported	Reported
Turek	Reported	Addressed by exclusions	Asthma and cancer status addressed	Not reported	Reported

## Data Availability

The data supporting the findings of this systematic review and meta-analysis are included in the article and Supplementary Materials, including the extracted study characteristics, diversity data, taxa abundance data, risk of bias assessments, search strategies, and excluded-study records. The source data were derived from previously published studies cited in the manuscript. Further inquiries can be directed to the corresponding author.

## Supplementary Materials

The following supporting information can be downloaded at: https://www.mdpi.com/article/10.3390/microorganisms14081688/s1, Supplementary Material S1: PRISMA checklist. Supplementary Material S2: Search Queries for each database. Supplementary Material S3: Table S1, Study characteristics; Table S2, Alpha and Beta diversity data; Table S3, MINORS quality assessment. Supplementary Material S4: Taxa abundances for each airway niche. Supplementary Material S5: JBI risk of bias assessment checklist. Supplementary Material S6: Screening outcomes. Supplementary Material S7: Microbiome-specific methodological reporting. Supplementary Material S8: Table S4, Species-level findings per airway niche.

## Abbreviations

The following abbreviations are used in this manuscript:

16S rRNA	16S ribosomal RNA
ASV	Amplicon sequence variant
BAL	Bronchoalveolar lavage
BALF	Bronchoalveolar lavage fluid
CI	Confidence interval
COPD	Chronic obstructive pulmonary disease
Df	Degrees of freedom
DNA	Deoxyribonucleic acid
EC	Electronic cigarette
ENDS	Electronic Nicotine Delivery Systems
ETS	Environmental Tobacco Smoke
I^2^	I-squared heterogeneity statistic
MeSH	Medical Subject Headings
MINORS	Methodological index for Non-randomized Studies
MyD88	Myeloid differentiation primary response 88
NELF	Nasal epithelial lining fluid
OTUs	Operational taxonomic units
PICO	Population, Intervention/Exposure, Comparator, Outcome
PRISMA	Preferred reporting items for systematic reviews and meta-analyses
REML	Restricted maximum likelihood
RevMan	Review Manager
SMDs	Standardized mean differences
URT	Upper respiratory tract
Z	Z statistic
χ^2^	Chi-square statistic
τ^2^	Tau-squared between-study variance
