# Supplementary material for: The Effects of Smoking on Respiratory Microbiota Diversity and Composition: A Systematic Review with Exploratory Meta-Analysis
Source: Microorganisms. 2026 Aug 1;14(8):1688. doi: 10.3390/microorganisms14081688 (PMC13515813; doi:10.3390/microorganisms14081688)
Supplement: Supplementary file 1 [file microorganisms-14-01688-s001.zip › Supplementary Material S8.pdf]

Supplementary Material S8

Table S4: This table shows the key species-level findings from the core articles in this systematic review.

Site	Study	Enriched Species	Depleted Species
**Nasal**	Hickman et al., 2024	*Staphylococcus Aureus*	*Cutibacterium acnes*
	Paulo et al., 2023	*Rothia dentocariosa*<br>*Prevotella melaninogenica*<br>*Veillonella atypica*	*Haemophilus parahaemolyticus/H. sputorum*<br>*Corynebacterium propinquum/C. pseudodiphtheriticum*<br>*Dolosigranulum pigrum/uncultured Alloiococcus spp. (ASV72)*<br>*Streptococcus sanguinis*
	Pfeiffer et al., 2022	*Staphylococcus epidermidis*<br>*Propionibacterium sp.*	*Dolosigranulum pigrum*
**Upper**	Turek et al., 2021	*Streptococcus parasanguinis*<br>*S. mitis/pneumoniae*<br>*S. salivarius*<br>*S. thermophilus*	*Streptococcus oralis*<br>*Streptococcus sp. I-G2*
	Ying et al., 2022	*Lactobacillus gasseri*<br>*Lactobacillus fermentum*<br>*Streptococcus lutetiensis*<br>*Lactobacillus crispatus*<br>*Lactobacillus helveticus*	
	Paulo et al., 2023	*Selenomonas sputigena*<br>*Bacillus spp.*<br>*Burkholderia spp.*	*Campylobacter showae/C. rectus*<br>*Leptotrichia spp.*
	Pfeiffer et al., 2022		*Centipeda periodontii*
**Lower**	Ying et al., 2022	*Niastella koreensis*<br>*Polaribacter sp. SA4-12*<br>*Myroides odoratimimus*<br>*Vibrio tasmaniensis*<br>*Paraburkholderia rhizoxinica*<br>*Stigmatella aurantiaca*<br>*Lactobacillus kefiranofaciens*<br>*Paraburkholderia terrae*	*Neisseria elongata*<br>*Neisseria sicca*<br>*Haemophilus parainfluenzae*
	Pfeiffer et al., 2022	*Acinetobacter bereziniae*<br>*A. johnsonii*<br>*Cupriavidus metallidurans*<br>*Serratia marcescens*	*Prevotella and Veillonella species*

		Stenotrophomonas maltophilia	
