# Supplementary material for: The Effects of Smoking on Respiratory Microbiota Diversity and Composition: A Systematic Review with Exploratory Meta-Analysis
Source: Microorganisms. 2026 Aug 1;14(8):1688. doi: 10.3390/microorganisms14081688 (PMC13515813; doi:10.3390/microorganisms14081688)
Supplement: Supplementary file 1 [file microorganisms-14-01688-s001.zip › Supplementary Material S2.pdf]

Supplementary Material S2

Queries used for article search.

PUBMED

("Respiratory Tract"[MeSH] OR "Respiratory Mucosa"[MeSH] OR "Mucosa, Respiratory" OR "Respiratory Epithelium" OR "Epithelium, Respiratory" OR "Lung"[MeSH] OR “Lungs” OR "Nasal Cavity"[MeSH] OR "Noses" OR "External Nose" OR "External Noses" OR "Nose, External" OR "Noses, External" OR "Cavities, Nasal" OR "Cavity, Nasal" OR "Nasal Cavities" OR "Mucosa, Nasal" OR "Nasal Epithelium" OR "Epithelium, Nasal" OR "Schneiderian Membrane" OR "Membrane, Schneiderian" OR "Pharynx" OR "Throat" OR "Nasopharynx"[MeSH] OR "Hypopharynx" OR "Laryngopharynx" OR "Oropharynx" OR "Larynx"[MeSH] OR "Laryngeal Anterior Commissure" OR "Anterior Commissures, Laryngeal" OR "Commissure, Laryngeal Anterior" OR "Commissures, Laryngeal Anterior" OR "Laryngeal Anterior Commissures" OR "Anterior Commissure, Laryngeal" OR "Anterior Commissure, Larynx" OR "Anterior Commissures, Larynx" OR "Commissure, Larynx Anterior" OR "Commissures, Larynx Anterior" OR "Larynx Anterior Commissure" OR "Larynx Anterior Commissures" OR "Laryngeal Posterior Commissure" OR "Commissure, Laryngeal Posterior" OR "Commissures, Laryngeal Posterior" OR "Laryngeal Posterior Commissures" OR "Posterior Commissures, Laryngeal" OR "Posterior Commissure, Laryngeal" OR "Posterior Commissure, Larynx" OR "Commissure, Larynx Posterior" OR "Commissures, Larynx Posterior" OR "Larynx Posterior Commissure" OR "Larynx Posterior Commissures" OR "Posterior Commissures, Larynx" OR “Middle meatus” OR “Sinus, Maxillary” OR “Antrum of Highmore” OR “Highmore Antrum” OR “Maxillary Antrum” OR “Antrum, Maxillary” OR “Maxillary Antrums” OR “Maxillary Ostium” OR “Maxillary Ostiums” OR "Trachea"[MeSH] OR “Tracheas” OR "Bronchi"[MeSH] OR “Bronchus” OR “Primary Bronchi” OR “Bronchi, Primary” OR “Bronchus, Primary” OR “Primary Bronchus” OR“Secondary Bronchi” OR “Bronchi, Secondary” OR “Bronchus, Secondary” OR “Secondary Bronchus” OR “Tertiary Bronchi” OR “Bronchi, Tertiary” OR “Bronchus, Tertiary” OR “Tertiary Bronchus” OR "Pulmonary Alveoli"[MeSH] OR “Alveoli, Pulmonary” OR “Alveolus, Pulmonary” OR “Pulmonary Alveolus”)

AND

("Microbiota"[MeSH] OR "Bacteria"[MeSH] OR "Human Microbiome"[MeSH] OR “Microbiome” OR “Microbiomes” OR “human microbiome” OR “human microbiomes” OR “Microbiotas” OR "Microbial Community" OR "Community, Microbial" OR "Microbial Communities" OR "Microbial Community Composition" OR "Community Composition,

Microbial" OR "Composition, Microbial Community" OR "Microbial Community Compositions" OR "Microbial Community Structure" OR "Community Structure, Microbial" OR "Microbial Community Structures")

AND

("Smoking"[MeSH] OR "Smoking Behaviors"[MeSH] OR "Behavior, Smoking" OR "Behaviors, Smoking" OR "Smoking Behavior" OR "Smoking Habit" OR "Habit, Smoking" OR "Habits, Smoking" OR "Smoking Habits" OR "Smoking, Tobacco" OR "Smoking, Pipe" OR "Pipe Smoking, Water" OR "Smoking, Water Pipe" OR "Waterpipe Smoking" OR "Smoking, Waterpipe" OR "Hookah Smoking" OR "Smoking, Hookah" OR "Smoking, Cigarette" OR "Smoking, Cigar" OR "THC Vaping" OR "THC Vapings" OR "Vapings, THC" OR "Vaping, THC" OR "Ecigarette Use" OR "Ecigarette Uses" OR "Use, Ecigarette" OR "Uses, Ecigarette" OR "ECig Use" OR "ECig Uses" OR "Use, ECig" OR "Vape" OR "Vapes" OR "E-Cigarette Use" OR "E Cigarette Use" OR "E-Cigarette Uses" OR "Use, E-Cigarette" OR "Nicotine Vaping" OR "Nicotine Vapings" OR "Vaping, Nicotine" OR "Vapings, Nicotine" OR "E-Cig Use" OR "E Cig Use" OR "E-Cig Uses" OR "Use, E-Cig" OR "Electronic Cigarette Use" OR "Cigarette Use, Electronic" OR "Electronic Cigarette Uses" OR "Use, Electronic Cigarette" OR "Smoker" OR "Smokers, Tobacco" OR "Smoker, Tobacco" OR "Tobacco Smoker" OR "Tobacco Smokers" OR "Smokers, Non-Tobacco Products" OR "Non-Tobacco Products Smoker" OR "Non-Tobacco Products Smokers" OR "Smoker, Non-Tobacco Products" OR "Smokers, Non Tobacco Products" OR "Vapers" OR "Vaper" OR "Electronic Nicotine Delivery System" OR "Electronic Cigarettes" OR "Electronic Cigarette" OR "Cigarette, Electronic" OR "Cigarettes, Electronic" OR "E-Cigs" OR "E Cigs" OR "E-Cigarettes" OR "E Cigarettes" OR "E-Cigarette" OR "E Cigarette" OR "E-Cig" OR "E Cig")

Scopus

TITLE-ABS-KEY(("Respiratory Tract" OR "Respiratory Mucosa" OR "Mucosa, Respiratory" OR "Respiratory Epithelium" OR "Epithelium, Respiratory" OR "Lung" OR "Lungs" OR "Nasal Cavity" OR "Noses" OR "External Nose" OR "External Noses" OR "Nose, External" OR "Noses, External" OR "Cavities, Nasal" OR "Cavity, Nasal" OR "Nasal Cavities" OR "Mucosa, Nasal" OR "Nasal Epithelium" OR "Epithelium, Nasal" OR "Schneiderian Membrane" OR "Membrane, Schneiderian" OR "Pharynx" OR "Throat" OR "Nasopharynx" OR "Hypopharynx" OR "Laryngopharynx" OR "Oropharynx" OR "Larynx" OR "Laryngeal Anterior Commissure" OR "Anterior Commissures, Laryngeal" OR "Commissure, Laryngeal Anterior" OR "Commissures, Laryngeal Anterior" OR "Laryngeal Anterior Commissures" OR "Anterior Commissure, Laryngeal" OR "Anterior Commissure, Larynx" OR "Anterior Commissures, Larynx" OR "Commissure, Larynx Anterior" OR "Commissures, Larynx Anterior" OR "Larynx Anterior Commissure" OR "Larynx Anterior Commissures" OR "Laryngeal Posterior Commissure" OR "Commissure, Laryngeal Posterior" OR "Commissures, Laryngeal Posterior"

OR "Laryngeal Posterior Commissures" OR "Posterior Commissures, Laryngeal" OR "Posterior Commissure, Laryngeal" OR "Posterior Commissure, Larynx" OR "Commissure, Larynx Posterior" OR "Commissures, Larynx Posterior" OR "Larynx Posterior Commissure" OR "Larynx Posterior Commissures" OR "Posterior Commissures, Larynx" OR "Middle meatus" OR "Sinus, Maxillary" OR "Antrum of Highmore" OR "Highmore Antrum" OR "Maxillary Antrum" OR "Antrum, Maxillary" OR "Maxillary Antrums" OR "Maxillary Ostium" OR "Maxillary Ostiums" OR "Trachea" OR "Tracheas" OR "Bronchi" OR "Bronchus" OR "Primary Bronchi" OR "Bronchi, Primary" OR "Bronchus, Primary" OR "Primary Bronchus" OR "Secondary Bronchi" OR "Bronchi, Secondary" OR "Bronchus, Secondary" OR "Secondary Bronchus" OR "Tertiary Bronchi" OR "Bronchi, Tertiary" OR "Bronchus, Tertiary" OR "Tertiary Bronchus" OR "Pulmonary Alveoli" OR "Alveoli, Pulmonary" OR "Alveolus, Pulmonary" OR "Pulmonary Alveolus")) AND TITLE-ABS-KEY(("Microbiota" OR "Bacteria" OR "Human Microbiome" OR "Microbiome" OR "Microbiomes" OR "human microbiome" OR "human microbiomes" OR "Microbiotas" OR "Microbial Community" OR "Community, Microbial" OR "Microbial Communities" OR "Microbial Community Composition" OR "Community Composition, Microbial" OR "Composition, Microbial Community" OR "Microbial Community Compositions" OR "Microbial Community Structure" OR "Community Structure, Microbial" OR "Microbial Community Structures")) AND TITLE-ABS-KEY(("Smoking" OR "Smoking Behaviors" OR "Behavior, Smoking" OR "Behaviors, Smoking" OR "Smoking Behavior" OR "Smoking Habit" OR "Habit, Smoking" OR "Habits, Smoking" OR "Smoking Habits" OR "Smoking, Tobacco" OR "Smoking, Pipe" OR "Pipe Smoking, Water" OR "Smoking, Water Pipe" OR "Waterpipe Smoking" OR "Smoking, Waterpipe" OR "Hookah Smoking" OR "Smoking, Hookah" OR "Smoking, Cigarette" OR "Smoking, Cigar" OR "THC Vaping" OR "THC Vapings" OR "Vapings, THC" OR "Vaping, THC" OR "Ecigarette Use" OR "Ecigarette Uses" OR "Use, Ecigarette" OR "Uses, Ecigarette" OR "ECig Use" OR "ECig Uses" OR "Use, ECig" OR "Vape" OR "Vapes" OR "E-Cigarette Use" OR "E Cigarette Use" OR "E-Cigarette Uses" OR "Use, E-Cigarette" OR "Nicotine Vaping" OR "Nicotine Vapings" OR "Vaping, Nicotine" OR "Vapings, Nicotine" OR "E-Cig Use" OR "E Cig Use" OR "E-Cig Uses" OR "Use, E-Cig" OR "Electronic Cigarette Use" OR "Cigarette Use, Electronic" OR "Electronic Cigarette Uses" OR "Use, Electronic Cigarette" OR "Smoker" OR "Smokers, Tobacco" OR "Smoker, Tobacco" OR "Tobacco Smoker" OR "Tobacco Smokers" OR "Smokers, Non-Tobacco Products" OR "Non-Tobacco Products Smoker" OR "Non-Tobacco Products Smokers" OR "Smoker, Non-Tobacco Products" OR "Smokers, Non Tobacco Products" OR "Vapers" OR "Vaper" OR "Electronic Nicotine Delivery System" OR "Electronic Cigarettes" OR "Electronic Cigarette" OR "Cigarette, Electronic" OR "Cigarettes, Electronic" OR "E-Cigs" OR "E Cigs" OR "E-Cigarettes" OR "E Cigarettes" OR "E-Cigarette" OR "E Cigarette" OR "E-Cig" OR "E Cig"))

Web of Science

TS=("Respiratory Tract" OR "Respiratory Mucosa" OR "Mucosa, Respiratory" OR "Respiratory Epithelium" OR "Epithelium, Respiratory" OR "Lung" OR "Lungs" OR "Nasal Cavity" OR "Noses" OR "External Nose" OR "External Noses" OR "Nose, External" OR "Noses, External" OR "Cavities, Nasal" OR "Cavity, Nasal" OR "Nasal Cavities" OR "Mucosa, Nasal" OR "Nasal Epithelium" OR "Epithelium, Nasal" OR "Schneiderian Membrane" OR "Membrane, Schneiderian" OR "Pharynx" OR "Throat" OR "Nasopharynx" OR "Hypopharynx" OR "Laryngopharynx" OR "Oropharynx" OR "Larynx" OR "Laryngeal Anterior Commissure" OR "Anterior Commissures, Laryngeal" OR "Commissure, Laryngeal Anterior" OR "Commissures, Laryngeal Anterior" OR "Laryngeal Anterior Commissures" OR "Anterior Commissure, Laryngeal" OR "Anterior Commissure, Larynx" OR "Anterior Commissures, Larynx" OR "Commissure, Larynx Anterior" OR "Commissures, Larynx Anterior" OR "Larynx Anterior Commissure" OR "Larynx Anterior Commissures" OR "Laryngeal Posterior Commissure" OR "Commissure, Laryngeal Posterior" OR "Commissures, Laryngeal Posterior" OR "Laryngeal Posterior Commissures" OR "Posterior Commissures, Laryngeal" OR "Posterior Commissure, Laryngeal" OR "Posterior Commissure, Larynx" OR "Commissure, Larynx Posterior" OR "Commissures, Larynx Posterior" OR "Larynx Posterior Commissure" OR "Larynx Posterior Commissures" OR "Posterior Commissures, Larynx" OR "Middle meatus" OR "Sinus, Maxillary" OR "Antrum of Highmore" OR "Highmore Antrum" OR "Maxillary Antrum" OR "Antrum, Maxillary" OR "Maxillary Antrums" OR "Maxillary Ostium" OR "Maxillary Ostiums" OR "Trachea" OR "Tracheas" OR "Bronchi" OR "Bronchus" OR "Primary Bronchi" OR "Bronchi, Primary" OR "Bronchus, Primary" OR "Primary Bronchus" OR "Secondary Bronchi" OR "Bronchi, Secondary" OR "Bronchus, Secondary" OR "Secondary Bronchus" OR "Tertiary Bronchi" OR "Bronchi, Tertiary" OR "Bronchus, Tertiary" OR "Tertiary Bronchus" OR "Pulmonary Alveoli" OR "Alveoli, Pulmonary" OR "Alveolus, Pulmonary" OR "Pulmonary Alveolus")

AND

TS=("Microbiota" OR "Bacteria" OR "Human Microbiome" OR "Microbiome" OR "Microbiomes" OR "human microbiome" OR "human microbiomes" OR "Microbiotas" OR "Microbial Community" OR "Community, Microbial" OR "Microbial Communities" OR "Microbial Community Composition" OR "Community Composition, Microbial" OR "Composition, Microbial Community" OR "Microbial Community Compositions" OR "Microbial Community Structure" OR "Community Structure, Microbial" OR "Microbial Community Structures")

AND

TS =("Smoking" OR "Smoking Behaviors" OR "Behavior, Smoking" OR "Behaviors, Smoking" OR "Smoking Behavior" OR "Smoking Habit" OR "Habit, Smoking" OR "Habits, Smoking" OR "Smoking Habits" OR "Smoking, Tobacco" OR "Smoking, Pipe" OR "Pipe Smoking, Water" OR

"Smoking, Water Pipe" OR "Waterpipe Smoking" OR "Smoking, Waterpipe" OR "Hookah Smoking" OR "Smoking, Hookah" OR "Smoking, Cigarette" OR "Smoking, Cigar" OR "THC Vaping" OR "THC Vapings" OR "Vapings, THC" OR "Vaping, THC" OR "Ecigarette Use" OR "Ecigarette Uses" OR "Use, Ecigarette" OR "Uses, Ecigarette" OR "ECig Use" OR "ECig Uses" OR "Use, ECig" OR "Vape" OR "Vapes" OR "E-Cigarette Use" OR "E Cigarette Use" OR "E-Cigarette Uses" OR "Use, E-Cigarette" OR "Nicotine Vaping" OR "Nicotine Vapings" OR "Vaping, Nicotine" OR "Vapings, Nicotine" OR "E-Cig Use" OR "E Cig Use" OR "E-Cig Uses" OR "Use, E-Cig" OR "Electronic Cigarette Use" OR "Cigarette Use, Electronic" OR "Electronic Cigarette Uses" OR "Use, Electronic Cigarette" OR "Smoker" OR "Smokers, Tobacco" OR "Smoker, Tobacco" OR "Tobacco Smoker" OR "Tobacco Smokers" OR "Smokers, Non-Tobacco Products" OR "Non-Tobacco Products Smoker" OR "Non-Tobacco Products Smokers" OR "Smoker, Non-Tobacco Products" OR "Smokers, Non Tobacco Products" OR "Vapers" OR "Vaper" OR "Electronic Nicotine Delivery System" OR "Electronic Cigarettes" OR "Electronic Cigarette" OR "Cigarette, Electronic" OR "Cigarettes, Electronic" OR "E-Cigs" OR "E Cigs" OR "E-Cigarettes" OR "E Cigarettes" OR "E-Cigarette" OR "E Cigarette" OR "E-Cig" OR "E Cig")
