# Supplementary material for: The Effects of Smoking on Respiratory Microbiota Diversity and Composition: A Systematic Review with Exploratory Meta-Analysis
Source: Microorganisms. 2026 Aug 1;14(8):1688. doi: 10.3390/microorganisms14081688 (PMC13515813; doi:10.3390/microorganisms14081688)
Supplement: Supplementary file 1 [file microorganisms-14-01688-s001.zip › Supplementary Material S3.pdf]

Supplementary Material S3

Table S1: This table summarizes the characteristics of each study included in the systematic review such as study design, country, study groups and size and population description.

Article	Country	Study Design	Study Groups and their size	Population Description
Hickman et al., 2024	USA	Original Investigation; human subjects with single-visit sampling (2014–2018) comparing nonsmokers, smokers, and e-cigarette users	Microbiome analyses: N=62 (19 nonsmokers, 19 smokers, 24 e-cigarette users). NLF mediator analyses: N=66 (20 nonsmokers, 19 smokers, 27 e-cigarette users)	Healthy adults aged 18–50 years; excluded asthma, COPD, cardiorespiratory disease, nasal surgery, immunodeficiency, pregnancy, etc. E-cigarette users were younger on average than nonsmokers and smokers
Pfeiffer et al., 2022	Germany	healthy never-smokers, ex-smokers, and current smokers; smokers stratified by intensity/duration (mild, short-term heavy, long-term heavy).	Total enrolled n=58. Sampling: nasal n=46; oropharyngeal n=54; bronchoalveolar lavage (BAL) n=52. Nasal smoking-status subgroups: never n=10, ex n=6, smokers n=30. Oropharyngeal: never n=14, smokers n=34, ex n=6. Lung figure compares never-smokers vs smokers within n=52.	Healthy adults. Active/passive exposure categorised by urine nicotine, cotinine, 3-OH-cotinine, anabasine (LC-MS/MS). Smokers stratified as: long-term (≥10 cigarettes/day and ≥10 pack-years), short-term heavy (≥10/day and <10 pack-years), mild (<10/day and <5 pack-years).
Paulo et al., 2023	Portugal	Case–control study ; paired nasopharyngeal and oropharyngeal samples; analysis adjusted for age, gender, contact with children, smoking, and season.	N=59 adults (12 pneumococcal carriers; 47 noncarriers). Smoking status: 25 smokers, 34 non-smokers.	Immunocompetent healthy adults aged 25–50 years, living in Portugal. Samples within 1 month of antibiotic use excluded
Bach et al., 2023	United Kingdom	Longitudinal weekly oropharyngeal sampling over ~30–40 weeks; smokers vs nonsmokers	N=30 participants: 18 nonsmokers, 12 smokers; 21 females, 9 males (10 female and 2 male smokers).	Healthy adults aged 18–40 years.
Campos et al., 2023	USA	Prospective single-centre cohort; bronchoalveolar lavage (BAL) from never smokers (NS), former smokers (FS), active smokers (AS);	Total n=55 (NS n=9; FS n=24; AS n=22).	>40 years; smokers ≥10 pack-years. FS abstinent ≥12 months; AS smoked within 3 days.
Liu et al., 2022	China	Observational study using bronchoalveolar lavage (BAL) to compare smokers vs. non-smokers; metagenomic NGS of microbial DNA; group comparisons of diversity and composition.	Total n=55; non-smokers n=42; smokers n=13. Among smokers: median 18 pack-years (range 7–41). Male/Female: 37/18.	Subjects undergoing bronchoscopy
Gioula et al., 2018	Greece	Prospective study of healthy volunteers with subgroup analyses (including smoking status); single pharyngeal swab per subject; 16S rRNA deep sequencing and community analyses.	Total N=10 healthy volunteers; smokers n=3; non-smokers n=7.	Greek nationality; No influenza vaccination or no respiratory infection in prior year; no antibiotics in prior 6 months.
Jetté, 2016	USA	Healthy laryngeal biopsies; smoking/reflux groups	Pyrosequenced N=97 total (77 nonsmokers; 20 smokers). Reflux strata within nonsmokers: GERD n=19, LPR n=28, Normal n=30; within smokers: GERD n=10, LPR n=6, Normal n=4. Overall mean age 42 (range 21–65); 56% female.	Healthy volunteers aged 21–65, recruited in Madison, WI; Exclusions included prior head/neck radiation (≤5y), certain surgeries, recent infectious laryngitis (<3 mo), chronic sinusitis/rhinitis (≤1 y), use of PPIs/H2 blockers in the past year, steroids, immunomodulators, high alcohol use, pregnancy/lactation. Nonsmokers had not smoked within prior year; smokers ≥5 cigarettes/day (≥1 year).

Ying et al., 2022	USA	Cross-sectional study of healthy adults comparing never-smokers, electronic cigarette (EC) users, and smokers; bronchoalveolar lavage (BAL) and saliva underwent metatranscriptome RNA-seq; pairwise smoking-status comparisons (SM vs NS, SM vs EC, EC vs NS).	Total n=28 (NS n=10, EC n=10, SM n=8). BAL collected for all 28 (one SM lung sample removed after QC); saliva n=26 (two failed sequencing).	Healthy adults aged 21–30 y (mean ~26 y); NS <100 lifetime cigarettes and no EC ≥1 y; EC users daily/frequent ≥6 mo and no cigarettes ≥6 mo (8/10 were former smokers); SM ≥100 lifetime cigarettes and ≥5 cig/day for ≥6 mo; bronchoscopy performed; demographics roughly balanced by age/sex/race across groups.
Morris et al., 2013	USA	Prospective, observational cohort comparing oral wash (OW) and bronchoalveolar lavage (BAL) from healthy nonsmokers vs smokers; applied a neutral community model to identify lung-enriched taxa.	N=64 adults: nonsmokers n=45, smokers n=19. Smokers median pack-years 18 (range 7–41).	Healthy HIV-uninfected adults aged 18–80; no antibiotics (3 mo) or immunosuppressants (6 mo); no acute respiratory symptoms in prior 4 wks; excluded any pulmonary disease or abnormal PFT. Participants fasted and refrained from smoking ≥12 h before sampling.
Yu et al., 2017	USA	Cross-sectional comparison of current smokers vs never-smokers across eight oral subsites and anterior nares; nine samples per person; 16S rRNA on Illumina MiSeq.	N=43 adults: current smokers n=23; never-smokers n=20. Nine specimens targeted per subject (some subgingival samples failed sequencing).	Adults recruited at Eastman Institute of Oral Health (Rochester, NY). Smokers: median 15 y duration; median 15 cigarettes/day. Never-smokers: <100 lifetime cigarettes. Exclusions: antibiotics or professional dental cleaning within 3 months; periodontal disease; cancer; >1 tooth lost. Groups frequency-matched by gender and race; IRB-approved with informed consent.
Charlson et al., 2010	USA	Cross-sectional comparison of healthy adult smokers vs. nonsmokers with paired right/left nasopharyngeal and oropharyngeal swabs; culture-independent 16S rRNA deep sequencing	N=62 participants: smokers n=29; nonsmokers n=33. A subset was sampled more than once (n=6: 5 smokers, 1 nonsmoker).	Healthy, asymptomatic adults recruited in Philadelphia, no antibiotics in prior 3 months; smokers >2 cigarettes/day for >6 months; nonsmokers <100 lifetime cigarettes; median time from last cigarette in smokers 1.5 h (range 1 min–21 h).
Turek et al., 2021	Australia	Cross-sectional general population study of adults with posterior oropharyngeal swabs; 16S rRNA profiling and network (WGCNA) analysis; ;comparisons by smoking status and asthma.	Samples passing QC: n=529. Current smokers n=60; ex-smokers n=216; never-smokers n≈253. Asthmatics n=77 (35% on ICS). For some analyses, a smoking subgroup with pack-years >10 (n=159) was used.	Adults (mean age ~56 y) from the Busselton Healthy Ageing Study ("Baby Boomers" born 1946–1964); posterior oropharyngeal swabs collected beyond tonsils/palate; no antibiotics within 6 weeks; exclusions: diagnosed cancer; general Australian community sample.

Table S2: This table summarizes the main alpha and beta diversity findings of each study included in the systematic review.

Article	Specimen Type	Microbiome Analysis Method	Alpha Diversity Metrics	Beta Diversity Metrics
Hickman et al., 2024	Nasal epithelial lining fluid (NELF) strips for microbiome; nasal lavage fluid (NLF) for soluble mediator assays	16S rRNA gene sequencing (V3–V4) on Illumina MiSeq	Shannon, Simpson: No significant differences across exposure groups overall. Shannon higher in males vs females (p<0.05), significant within e-cigarette users.	UniFrac with PERMANOVA: no clear exposure-group separation; sex effect seen only in e-cigarette users.
Pfeiffer et al., 2022	Deep bilateral nasal swabs; bilateral oropharyngeal swabs; bronchoalveolar lavage (BAL).	16S rRNA sequencing (V3-V4)	Not reported	Not reported. Analyses focused on relative abundances and correlations with smoking metrics.
Paulo et al., 2023	Nasopharyngeal swabs and oropharyngeal swabs.	16S rRNA sequencing (V4, MiSeq)	Hill numbers (orders 0–4; relates to richness (q=0), Shannon (q=1), Simpson (q=2)). Nasopharynx: only marginal differences smokers vs non-smokers. Oropharynx: smokers showed significantly lower diversity	Aitchison distance with PERMANOVA: smoking significantly affected both nasopharyngeal and oropharyngeal communities.
Bach et al., 2023	Oropharyngeal swabs (tonsils/posterior pharyngeal wall)	16S rRNA sequencing (V1–V2).	Richness, Shannon, Simpson (Renyi–Hill). Healthy nonsmokers had significantly higher alpha diversity than healthy smokers; nonsmokers with colds showed lower alpha than healthy nonsmokers; antibiotics reduced diversity in both groups (except Simpson).	Bray-Curtis and UniFrac with PERMANOVA: smoking/health status significantly associated with community structure.
Campos et al., 2023	Bronchoalveolar lavage (BAL) from right middle lobe	16S rRNA sequencing (V6-V8)	Chao richness, inverse Simpson diversity, Faith's PD: lower diversity in former/current smokers than in never-smokers	UniFrac with PERMANOVA: groups separated, especially on unweighted UniFrac
Liu et al., 2022	Bronchoalveolar lavage fluid (BALF).	Metagenomic NGS	Shannon and Simpson: no significant difference between smokers and non-smokers.	PERMANOVA not reported; composition differences assessed via relative abundances and Wilcoxon tests (phylum/genus).
Gioula et al., 2018	Pharyngeal swab.	16S rRNA sequencing (Ion Torrent, V2-V9)	Richness and Shannon index: showed no significant differences.	Bray-Curtis with PCoA/PERMANOVA: significant smoking-related differences
Jetté et al., 2016	False vocal fold (laryngeal) tissue biopsies.	16S rRNA sequencing (454)	Smokers had significantly lower Shannon diversity than nonsmokers. Chao1 richness differed by reflux. No clear smoking-specific inverse Simpson signal	Bray-Curtis/Jaccard with PERMANOVA: significant differences by smoking status
Ying et al., 2022	BAL and saliva.	Meta-transcriptome RNA-seq	Lung (BAL): no between-group differences (Shannon). Oral (saliva): no between-group differences (Shannon), similar results with observed species and Gini-Simpson Index.	Lung: Bray–Curtis MDS showed NS and EC tending to cluster together vs SM, but PERMANOVA not significant. Oral: Bray–Curtis with PERMANOVA significant among groups, NS and EC cluster together vs SM.
Morris et al., 2013	Oral wash (OW) and BAL(right middle lobe or lingula)	16S rRNA gene sequencing on Roche 454 (V1-V3 and V3-V5).	V1–3: no significant effects of smoking status or site (OW vs BAL) after accounting for pairing. V3–5: observed OTUs higher in smokers (BAL and OW) vs nonsmokers (P=0.02). Shannon, inverse Simpson, PD: no additional smoking-related findings were reported	QYC-based comparisons: significant differences in OW (oral) communities between smokers and nonsmokers; no significant differences in BAL communities.
Yu, et al. 2017	Supra- and subgingival plaque scrapes; saliva; swabs from keratinized gingiva, hard palate, buccal mucosa, palatine tonsil, tongue dorsum; and an anterior nares (nasal) swab.	16S rRNA sequencing (V3-V4, MiSeq)	Across sites, no significant differences by smoking status except buccal mucosa: smokers showed lower alpha diversity (PD_whole_tree; Observed species no difference; Shannon nearly significant)	UniFrac with PERMANOVA: no significant smoking effect across sites
Charlson et al., 2010	Right and left nasopharyngeal swabs and oropharyngeal swabs.	16S rRNA sequencing on Roche 454 (V1-V2)	Shannon and Chao 1 index used; within each airway habitat, smokers were significantly more diverse than nonsmokers.	UniFrac/db-ANOVA: smoking separated oropharyngeal communities; nasopharyngeal differences were clearer on unweighted UniFrac

Turek et al., 2021	Posterior oropharyngeal swab	16S rRNA sequencing (V4, MiSeq)	Shannon diversity is significantly lower in current smokers than never-smokers. Diversity was independently related to current smoking and cumulative pack-years.	UniFrac/Bray-Curtis/WGCNA: no clear PERMANOVA signal but smoking strongly altered network structure.

Table S3: Methodological quality assessment of included studies using the MINORS tool. A total score (out of a maximum of 24) was calculated by adding the scores of each included study across the MINORS domains (e.g., clearly stated aim, prospective data collection, appropriate endpoints, control group adequacy, baseline equivalency, and statistical analysis). Each item was graded from 0 to 2. The mean MINORS score was 14.9 ± 1.2 (range 14–18) out of 24 across the 13 included comparative studies. This indicated some consistent methodological limitations across the studies.

Study	Year	Study type	Clearly stated aim	Inclusion of consecutive patients	Prospective collection of data	End points appropriate to study aim	Unbiased assessment of Study end point	Follow-Up Period Appropriate to Study Aim	Less Than 5% Lost to Follow Up	Prospective Calculation of the Study Size	Adequate control group	Contemporary Groups	Baseline equivalence of Groups	Adequate Statistical analysis	Total MINORS score
**Bach et al.**	2023	Comparative	2	0	2	2	1	2	0	0	2	1	1	2	15
**Campos et al.**	2023	Comparative	2	0	2	2	1	0	0	0	2	2	2	2	15
**Charlson et al.**	2010	Comparative	2	0	2	2	1	0	0	0	2	2	1	2	14
**Gioula et al.**	2018	Comparative	2	0	2	2	1	0	2	2	2	2	1	2	18
**Hickman et al.**	2025	Comparative	2	0	2	2	1	0	0	1	2	2	1	2	15
**Jette et al.**	2016	Comparative	2	0	2	2	1	0	0	0	2	2	1	2	14
**Liu et al.**	2022	Comparative	2	0	2	2	1	0	0	0	2	2	1	2	14
**Morris et al.**	2013	Comparative	2	0	2	2	1	0	0	0	2	2	1	2	14
**Paulo et al.**	2023	Comparative	2	0	2	2	1	2	0	0	2	2	1	2	16
**Pfeiffer et al.**	2022	Comparative	2	0	2	2	1	0	0	0	2	2	1	2	14
**Turek et al.**	2021	Comparative	2	0	2	2	1	0	0	2	2	2	1	2	16
**Ying et al.**	2022	Comparative	2	0	2	2	1	0	0	0	2	2	1	2	14
**Yu et al.**	2017	Comparative	2	0	2	2	1	0	0	0	2	2	2	2	15
